# Investigating coastal ecosystem health in Bangladesh using machine learning and remote sensing

**DOI:** 10.1371/journal.pone.0349170

**Published:** 2026-08-21

**Authors:** Angkon Roy, Showmitra Kumar Sarkar

**Affiliations:** 1 Department of Urban and Regional Planning, Khulna University of Engineering & Technology (KUET), Khulna, Bangladesh; 2 Department of Urban and Regional Planning, Khulna University of Engineering & Technology (KUET), Khulna, Bangladesh; Van Lang University: Truong Dai hoc Van Lang, VIET NAM

## Abstract

Ecosystem health is a measure that is used to describe the condition of the ecosystem. The stability of the ecosystem and sustainable growth are represented by ecosystem health which covers the overall condition of the environment. The loss of habitat and deterioration of ecosystem services are the outcomes of coastal urbanization and ecosystem health damage. Potential natural disasters and biodiversity loss are happening more frequently and intensely which makes an emerging issue of environmental resilience. That’s why ecosystem health assessment is must. The 1,47,570 km^2^ of Bangladesh has 47,201 km^2^ of coastal zone consisting of 19 districts which is 32% of the country and about 43.8 million people live here. This study aims to assess the ecosystem health of 2013 and 2023 and to investigate the change in the coastal ecosystem health in evaluating with past and present conditions of the ecosystem. For assessment, a random forest machine learning model has been used in conjunction with remote sensing and GIS due to its high accuracy and efficiencies. The PSR (Pressure-State-Response) model has been used in this investigation. This study reveals that the ecosystem health of 2023 deteriorated mostly than in 2013. About 19.76% of the lower ecosystem health area of coastal zone has been increased over the decades. In addition, 6.92% and 12.84% of higher and medium ecosystem health areas have been degraded accordingly. The Satkhira district had higher ecosystem health and Chittagong had lower ecosystem health in 2013; whereas in 2023, it became Narail and Jhalokathi accordingly. The point is that the ecosystem is down falling from the time on. This concerning factor can assist the body in making decisions on how should interact with the environment with adequate controlling crucial measures to heal the environment’s health.

## 1. Introduction

An ecological system is considered healthy and free from “distress syndrome” if it is stable and sustainable, which means that it is dynamic, resilient to stress, and keeps its organization and autonomy throughout time. Ecosystem health acts as an all-encompassing, dynamic, multiscale, hierarchical indicator of system vigor, resilience, and organization [1]. The essential components of ecosystem stability and sustainable growth are represented by ecosystem health, which can serve as a valuable guide for the superior development of urban agglomerations [2]. The country has 169.83 million people living in it and 1119 people per square kilometer [3]. Approximately 36 million individuals, or 29% of the total population, reside in the coastline region. Bangladesh’s coastline region is divided into 19 coastal districts [4]. The population density of this area is 743 people per square kilometer [5]. The coastline terrain is heavily utilized for tourism, industrial and infrastructure development, salt production, wetlands and fisheries, forestry, agriculture, and shrimp farms. Because they offer humans a variety of environmental products and services, coastal areas are significant from an ecological standpoint. They are home to important aquatic and terrestrial environments, including tidal flats, marshes, and mangrove forests. Char lands serve additional purposes [6]. Degradation of ecosystem services and habitat loss are direct results of coastal urbanization. Potential natural disasters, including storm surges, floods, droughts, and cyclones, will occur more frequently and with greater intensity. The coastal region of Bangladesh is more vulnerable to climate change because of its unique natural features, high population, and extreme poverty. Bangladesh’s coastal regions are particularly vulnerable in the west and center. The west is primarily made up of low-lying land tracts known as the Sundarbans, while the center is a more dynamic area [4]. The services provided by these ecosystems will be progressively reduced or lost as a result of climate change, with some effects perhaps being long-term [7].

Multiple studies have been conducted to evaluate the health of the ecosystem. [8] investigated the negative occurrences of Bangladesh’s southwest coastline region including the environmental degradation that is primarily caused by increased soil salinity, which is increased by a number of driving forces, including the production of fish and food and the conversion of rice to shrimp culture by analyzing a range of time series data of ecosystem services and drivers with environmental Kuznets curve analysis to understand the relation between economic growth and environmental protection. [9] undertaken a study on land-use change maps showing the alterations along the selected coast over the period 1989–2019 including how economic activity and land use change are co-evolving and changing the exposure of economic activities and human settlements and vulnerability and risk assessment of the Cox’s Bazar’s coastal ecosystem by the method of LULC mapping and questionnaire survey incorporating face to face interview. [10] assessed the study of influencing forces and ecosystem health in Inner Mongolia is characterized by the spatial heterogeneity of the ecosystem and nonstationary spatial connection correlation by using VORS model with Analytic Hierarchy Process (AHP) weighting method and Geographic Weighted Regression (GWR) model. [11] mapped the changes in LULC from 1990 to 2018 using SVM and use CA-ANN to predict LULC in 2028 while analyzing sensitivity (RF, CART, PDF, and Pearson’s). Create a fuzzy-based VORS model (1990–2028) to assess the health of ecosystems and used the Morris method sensitivity analysis to evaluate the performance of the EH model. [12] investigated the strengthen ecosystem service functions and advanced green development by enhancing the health of the forests in the WuZhi Shan region of the Hainan Tropical Rainforest National Park by using decision tree that is a machine learning approach and CRITIC method to calculate forest health. [9] investigated land-use change maps from 1989 to 2019 that illustrate the changes made along the chosen coast, how land use change and economic activity are interacting to alter how exposed human settlements are to economic activity and assessment of vulnerability and risk by LULC mapping and questionnaire survey. [13] Assessed the ecosystem health by using Principal Component Analysis (PCA) method. But none of the studies have not done using machine learning incorporating GIS and remote sensing on the coastal zone of Bangladesh to assess the ecosystem health though it has rich and complex system of biodiversity. Machine learning has high predictive accuracy with resistance to overfitting. It handles large datasets efficiently and robustness to noise and outlier. Also, they don’t use such kind of PSR framework incorporating GIS and remote sensing with machine learning techniques.

In this study, PSR (Pressure-State-Release) model has been considered. This is an effective technique that has many impact elements in addition, frequently employed in assessing the ecology [14,15]. Since the PSR approach asserts a distinct causal relationship between all the variables that are impacted, three groups of indicators are identified. Under the PSR framework, 20 factors have been considered in this investigation which everything is remotely sensing data. The random forest machine learning model has been used. The ecosystem health scenario and condition of 2013 and 2023 of coastal zone of Bangladesh has assessed that consisting of 19 districts has been chosen as study area. All the factors have gone through multi criteria analysis and fuzzification process using GIS for delivering the result. The machine learning model has been validated through ROC curve with higher accuracy that satisfied the model. This investigation reveals that the ecosystem health of 2023 has degraded mostly than 2013. About 19.76% of areas of low ecosystem health has been increased and 6.92% of areas of high ecosystem health has been decreased over the decade.

Therefore, the aim of this study is to assess the condition of the ecosystem health of 2013 and 2023 and to investigate the change of the coastal ecosystem health. That evaluates the past and present condition of the ecosystem health with changing patterns over the decades.

## 2. Methods and materials

### 2.1 Study area profile

**Fig 1:**
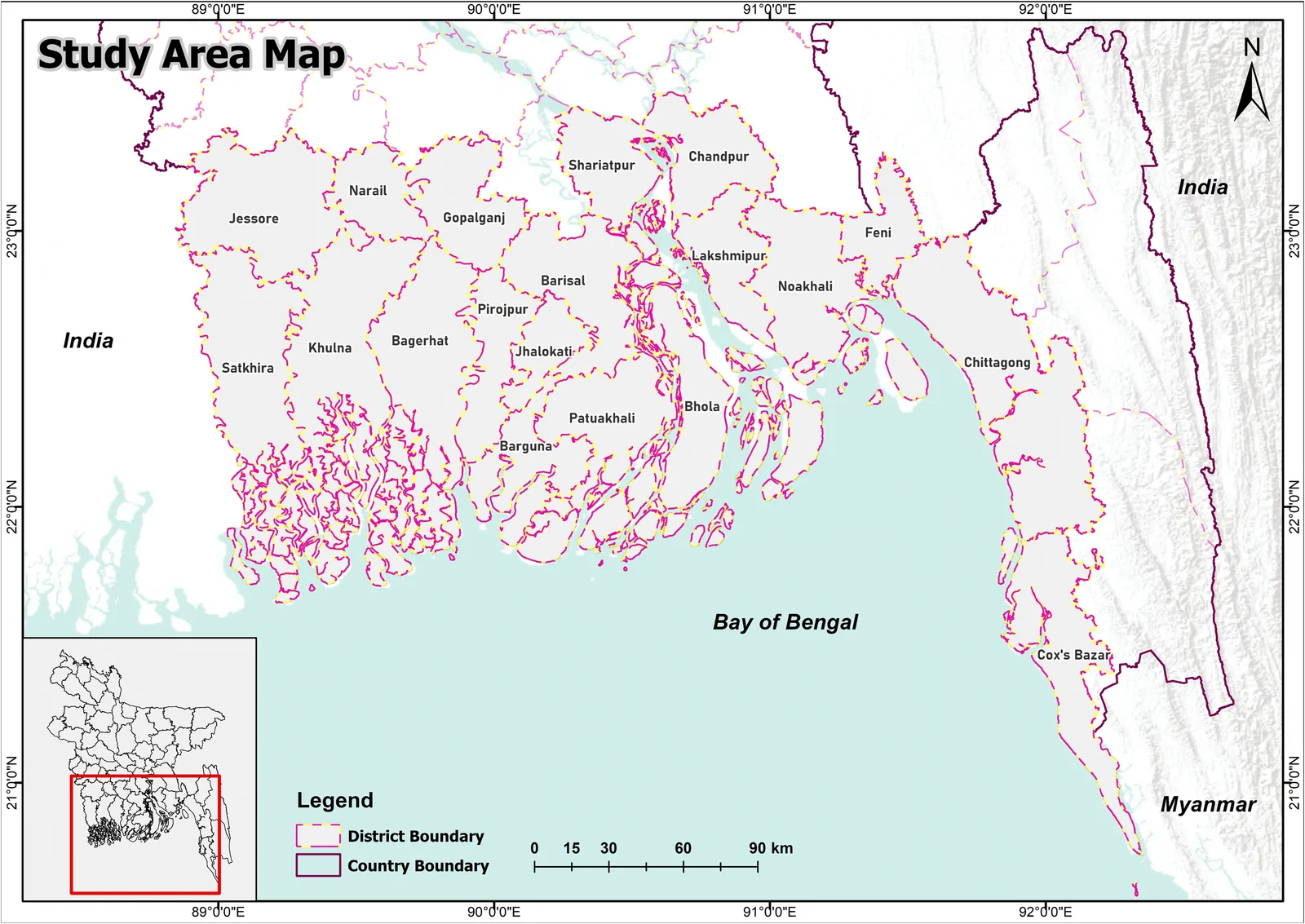
Study area. Extracted From: Bangladesh - Subnational Administrative Boundaries | Humanitarian Dataset | HDX.

The southern portion of Bangladesh (Fig 1) is home to the country’s coastal zone, which is endowed with a wealth of resources including mangroves, coral reefs, deltas, wetlands, saltmarshes, seaweeds, marine and coastal fisheries, flora and fauna, sea salts, beach minerals, sand dunes, and other natural features. From a geomorphological and hydrological perspective, the Bay of Bengal and the Ganges, Brahmaputra, and Meghna River systems dominate it. It is made up of 19 districts and occupies 47,201 km^2^, or 32% of the entire country. Over 43.8 million people call Bangladesh’s coastal regions home [5].

Bangladesh’s coastline extends 711 km and is made up of an extensive network of river systems that flow the Ganges, Brahmaputra, and Meghna River systems. [16]

### 2.2 Description of data

The EH of the coastal region of Bangladesh was analyzed by using the PSR (Pressure-State-Release) model. Under this model, various indicators were considered that can express the EH combinedly. A total of 20 indicators were considered according to the PSR model for the year 2013 and 2023 (Table 1). “Pressure” refers to forces that come from human activity. The purpose of the “State” indicators is to provide an overview of the current state of biological and functional quality and quantity, as well as any changes that have occurred over time. The quantity and kind of actions implemented by individuals and researchers are known as “Response” indicators. [15].

**Table 1:** List of the indicators of the study.

General Criteria	Indicators	Sources
**Pressure**	1. NDBI	Satellite Image (Landsat 8–9 OLI/TIRS C2 L2)
	2. Deforestation Index	Extracted from NDVI
	3. Cyclone prone areas	Regional Specialized Meteorological Centre (RSMC)
	4. Presence of air Pollutants (CO)	Giovanni, Earth Data (MOD05_L2 - MODIS/Terra Total Precipitable Water Vapor 5-Min L2 Swath 1 km and 5 km)
	5. Land Surface Temperature (LST)	(MOD11A1.061 Terra Land Surface Temperature and Emissivity Daily Global 1 km)
**State**	1. NDVI	Satellite Image (Landsat 8–9 OLI/TIRS C2 L2)
	2. NDWI	
	3. Soil pH	Soil Grids
	4. Precipitation	Bangladesh Agricultural Research Council (BARC)
	5. Temperature rate	
	6. Solar radiation	
	7. Humidity	
	8. Evaporation	Giovanni, Earth Data (MOD05_L2 - MODIS/Terra Total Precipitable Water Vapor 5-Min L2 Swath 1 km and 5 km)
	9. Surface wind speed	
	10. Ground water storage	
**Response**	1. Afforestation index	Extracted from NDVI
	2. Area ratio of natural reserve	[17]
	3. Spatially protected areas	
	4. Renewable energy potential areas	[18]
	5. Latent energy flux	Giovanni, Earth Data

A visual indicator called the NDBI Index is used to evaluate metropolitan areas, primarily built-up or artificially constructed places. Short Wave Infrared and Infrared Bands are the two main bands used in NDBI mapping. This index, which ranges from −1 to +1, is mostly used for built-up index mapping. A positive value denotes the presence of built-up areas or artificial structures, while a lower value denotes the presence of physical qualities. Roads and structures with higher NDBIs are examples of impermeable layers. Thus, the urban environment’s natural state and green ecosystem are negatively impacted by maximal NDBI values [19]. Deforestation, ecosystem services, and fragmentation, however, pose a growing threat to forests worldwide and have a substantial influence on human livelihoods, biodiversity, and ecosystem services. Significant environmental challenges that have garnered more attention in recent decades include changes in land use and forest deterioration. The main causes of these problems are human activities including industrialization, urbanization, and the growth of agriculture [20]. Global climate change, which may result in storms occurring more frequently and intensely, cyclones speeding up the rise of sea levels, and related economic hazards as well as the destruction of coastal habitats and natural resources. Which is an indicator of assessing EH [21]. Although carbon monoxide does not directly cause climate change, it can still have a significant impact on land and sea temperatures, alter entire ecosystems, and even create extreme weather conditions that increase the frequency of severe storms. Gases like carbon monoxide are easily linked to several climate change issues. Additionally, it may aid in the creation of tropospheric ozone, which turns into a secondary air pollutant [22]. As impermeable surfaces have a higher capacity to absorb and retain heat than natural surfaces, they are rapidly replacing natural surfaces in metropolitan areas, raising the land surface temperature (LST) relative to neighboring rural areas. Urban heat island (UHI) is the term used to describe the phenomena of overheating in urban and suburban areas. UHI has a negative effect on the environment and negatively affects urban ecology and biodiversity [23].

The values of the Normalized Difference Vegetation Index (NDVI) show whether or not there is vegetation in the area. It is computed using red visible and near-infrared bands. The index’s justification is that robust vegetation has a low reflectivity in the red and a high reflectance in the near-infrared (NIR), which improves the interpretation of plant cover while attenuating minor noise from other types of land cover [24]. The NDWI has values ranging from −1–1, with a positive value indicating the presence of waterbodies. High vegetation water content and high vegetation fraction cover are correlated with high NDWI results. Low vegetation water content and low vegetation fraction cover are correlated with low NDWI values [25]. Thus, in a tropical setting, evaluating the LST-NDWI relationship is crucial [26]. Soil pH has a direct and indirect impact on EH and it is a crucial factor. High pH causes soil toxicity, acidification and it provides soil fertility and productivity. pH is one of the key indicators of a soil’s ability to perform economic and environmental tasks. Certain characteristics are related to how temperature and rainfall variations brought about by a changing environment affect the pH of the soil [27]. Rainfall and the health of urban ecosystems are related, and their relationships are complex. Precipitation has a variety, if not detrimental, effects on the health of many urban sub-ecosystems [28]. Temperature produces heat and heat has a great significant effect on biodiversity and the ecosystem. It has various risk factors. It affects urban living. Enormous types of heat-related health risk is generated by temperature [29,30]. In the ecological, hydrological, and biophysical processes, solar radiation has a significant role. The total of the sun’s direct shortwave radiation and its dispersed radiation is known as solar radiation. As a result, in areas with sufficient water, the plant grows in response to increased solar radiation, which is seen as a positive indicator of the health of the ecosystem [31]. Variations in humidity have the potential to negatively impact ecosystem resilience to climate change as well as human and human health. The range of temperatures to which tropical ecosystems can adapt is far less, and these ecosystems are more vulnerable to the negative effects of rising humidity on ecosystems and human health [32]. The process of transforming a liquid phase into a gaseous phase at a temperature lower than its boiling point is called evaporation. Warm temperatures have the ability to accelerate evaporation and make surface water bodies under stress. The amount of moisture in the soil, evaporation, and precipitation are all impacted by atmospheric warming. The temperature rises cause more evaporation, which reduces outflow, whereas changes in the precipitation pattern brought on by climate change specifically increase precipitation and consequently outflow. This has an immediate impact on underground water availability and river flow. Climate change can also result in differences in seasonal precipitation and temperature shifts [33]. Surface wind speed, vegetation, precipitation, water resources, and surface temperature influence the rise in dust activity in the air and it affects the environment [34]. That’s why it is a potential factor. It creates hazards, transportation difficulties and health complexity. In addition, it directly affects the atmosphere. Groundwater storage tells the condition of the environment. Land holds water, vegetation holds land and water content in it and to the ground. Healthy ecosystem with potential interaction between vegetation, temperature, rainfall, land etc. increases the possibility to groundwater storage [35].

Natural heritage sites and the area ratio of natural reserves has an impact on EH. The landscape types and the population surrounding it and the overall environment of that area provide of that ecosystem [36]. Afforestation and reforestation policy has a great influence on assessing EH because afforestation makes the environment healthy and it can increase its healing capability [37]. Renewable energy means energy that may be used again and again, such as solar energy, wind power, hydroelectric power, geothermal energy from hot springs, tidal power from tides, biomass (biofuels), and the sun. Renewable energy causes less global warming, improves public health, creates economic benefits, stable energy prices and availability, reliability and resilience. A low carbon footprint has resulted from the provision of renewable energy supplies to lower carbon dioxide gas. As a result, they emit fewer greenhouse gases, which is good for the environment. Renewable energy sources generate thermal and electrical energy from natural resources such the sun, rivers, seas, air, and geothermal energy [38].

**Fig 2:**
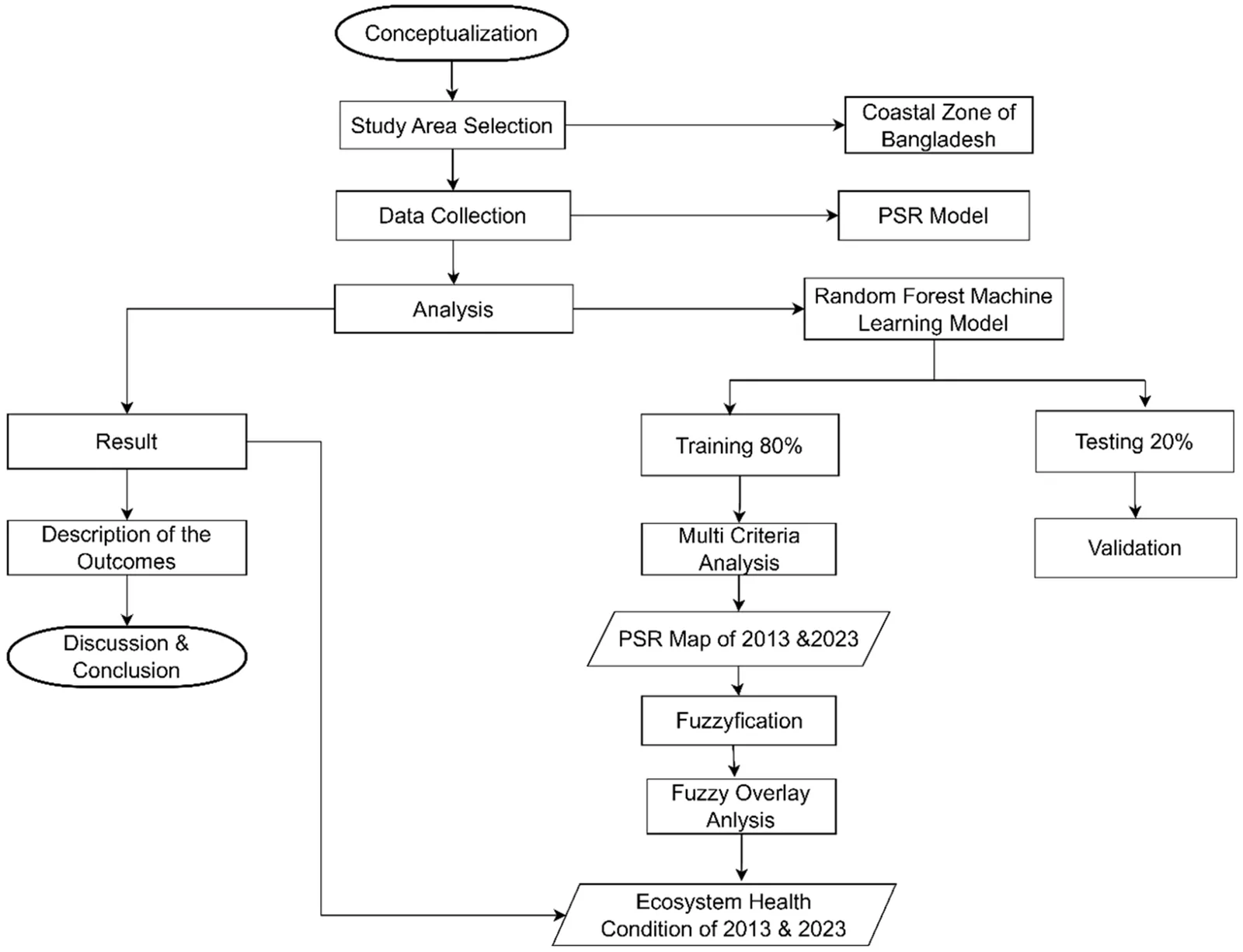
Methodological framework.

The energy that is transferred between the Earth’s surface and atmosphere during processes such as water vapor transpiring at the surface and then condensing in the troposphere is referred to as latent heat flux. Latent heat is related to phase transitions (such as solid to liquid, liquid to gas, or solid to gas) without changing the temperature or pressure of the substance involved, in contrast to sensible heat, which causes a temperature change. The methodological framework of this study is following (Fig 2):

### 2.3 Analytical method

#### 2.3.1 Random Forest machine learning model.

**Fig 3:**
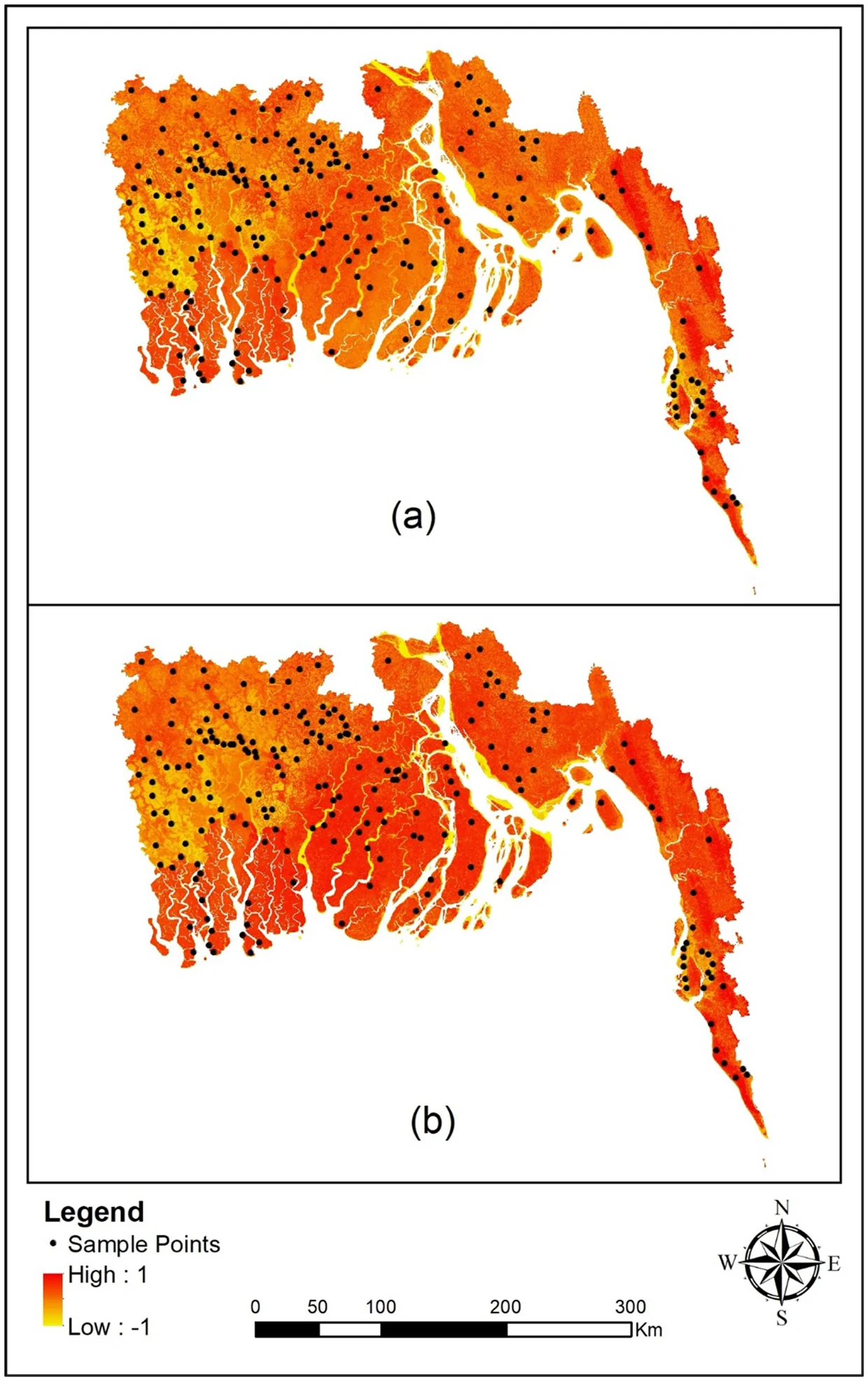
Sample points: (a) for 2013 and (b) for 2023. Extracted From: Bangladesh - Subnational Administrative Boundaries | Humanitarian Dataset | HDX.


The Random Forest was used for the sample randomly and it generates a decision tree. During the training stage, the decision trees were assessed independently. It constructed decision trees for every training data. The EH were determined for the year 2013 and 2023. In this investigation, 200 sample points were collected based on the Biodiversity Index map (Fig 3). The training data was 80% and the testing data was 20%. Those obtained data were processed through ArcGIS and a Random Forest machine learning algorithm was used. In this process, the data were normalized using an equation (Equation 1) [39].

$$\begin{array}{c} \text{X}=\frac{X-X_{min}}{X_{max}-X_{min}} \end{array}$$ (1)

#### 2.3.2 Multiple criteria analysis.

The indicators (Table 1) were used for multiple criteria analysis. MCA used the overlay approach which considered various layers which were the indicators to identify the suitable areas [40]. The weightage generated by machine learning model was the input of MCA for each indicator. The Pressure-State-Response was determined individually for 2013 and 2023 using ArcGIS 10.5. The output was reclassified using the natural breaks (Jenks) method into three classes of high, medium and low. The MCA gives the high, medium and low conditions of the EH according to the PSR model (Fig 12).

#### 2.3.3 Fuzzification and fuzzy overlay.

It is a process that transfers the input raster into a 0–1 scale. For fuzzy overlay analysis, the raster was transferred with this process (Fig 13). An extension of the indicator function in classical sets is the membership function of a fuzzy set. An element might be a part of a set or not in classical sets. The formula $\mu_{A\;}(X)\;$is true or false if (A) is a classic set. It depicts the degree of truth as an extension of valuation in fuzzy logic [41]. The “Membership Fuzzy” and “Fuzzy Overlay” operations located in the Spatial Analyst toolbox of ArcGIS software were used to complete the fuzzy model. The membership fuzzy tool offers a variety of linear and nonlinear fuzzy MF including large, small, Gaussian, linear, etc. [42]. In this study, “MSSmall” was used for all parameters. The mathematical expression is (Equation 2) [42]:


$$\begin{array}{c} {\text{SmallMF}\mu }_{\left(x\right)}=\frac{\text{1}}{\text{1}+{\left(\frac{x}{f_{\text{2}}}\right)}^{\frac{\text{1}}{\text{2}}}} \end{array}$$ (2)

All of the fuzzified raster inputs were combined using the fuzzy overlay technique. It has five distinct fuzzy combination operators: fuzzy OR, fuzzy AND, fuzzy Product, fuzzy Sum, and fuzzy Gamma. Whereas the overlay function “OR” chooses the highest value of all inputs, the overlay function “AND” chooses the lowest value of all inputs. All fuzzy inputs are multiplied by the “PRODUCT” function, resulting in a lower total evidence value than any one input. Since the inputs are merged linearly by the function “SUM,” the whole amount of evidence is more significant than any one input. Lastly, the fuzzy sum is multiplied by the fuzzy product to the power of gamma by the overlay function “GAMMA” [42,43]. This investigation uses “GAMMA” as overlay type with consideration of 90% of “GAMMA” (Equation 3) [43]. The ‘GAMMA” ranges from 0 to 1. By utilizing the ‘FUZZY-GAMMA” operation, the combination equals the fuzzy algebraic product when “GAMMA” is 0 and the fuzzy algebraic sum when “GAMMA” is 1. As a result, the membership combination can be optimized with the right “GAMMA” selection. Weights can be applied to many input values and combined into a single output value using overlay analysis tools. The general stages for solving a multicriteria problem are the same for all methods, despite their differences. Included in the fuzzy overlay analysis are two tools: “FUZZY MEMBERSHIP FUNCTIONS” which assign corresponding ratings for attribute values in a certain thematic layer between 0 and 1, and “FUZZY OVERLAY” which combines several “FUZZY MEMBERSHIP” results into the final composite map. To combine all of the input variables, a fuzzy “GAMMA” operation was performed with “GAMMA” = 0.9 since it resulted in the widest range of index values. When “GAMMA” ~ 0.9, more realistic zone spread can produce the most logical outcomes [44].

The overlay equations are:


$$\begin{array}{c} \text{Fuzzy AND Value}=\text{min }({\text{arg}}_{\text{1}},\ldots \ldots .,\;{\text{arg}}_{\text{n}}) \end{array}$$

$$\begin{array}{c} \text{Fuzzy OR Value}=\text{max }({\text{arg}}_{\text{1}},\ldots \ldots .,\;{\text{arg}}_{\text{n}}) \end{array}$$

$$\begin{array}{c} \text{Fuzzy Product Value}=\text{product }({\text{arg}}_{\text{1}},\ldots \ldots .,\;{\text{arg}}_{\text{n}}) \end{array}$$

$$\begin{array}{c} \text{Fuzzy Sum Value}=\text{1}\;-\text{ product }(\text{1}-{\text{arg}}_{\text{1}},\ldots \ldots .,\;\text{1}-{\text{arg}}_{\text{n}}) \end{array}$$

$$\begin{array}{c} \text{Fuzzy GAMMA Value}={\left(\text{FuzzySum}\right)}^{\lambda }*{\left(\text{FuzzySum}\right)}^{\text{1}-\lambda } \end{array}$$ (3)

#### 2.3.4 Validation of the model.

A ROC curve, also known as a receiver operating characteristic curve, is a graphical figure that shows how well a binary classifier model (which can also be used for multiclass analysis) performs at different threshold levels. An analysis based on true- and false-positive rates is provided by this curve [45]. By indicating the system’s accuracy in predicting the occurrence or non-occurrence of pre-defined events, the area under the ROC curve (AUC) describes the quality of a forecast system. The curve with the greatest AUC, which ranges from 0.5 to 1.0, is shown by the most perfect model. The AUC would be equal to 0.5 if the model could not forecast the EH any more accurately than a random approach. A perfect forecast has a ROC curve of 1 [46].

## 3. Result

### 3.1 Spatial distribution of data

**Fig 4:**
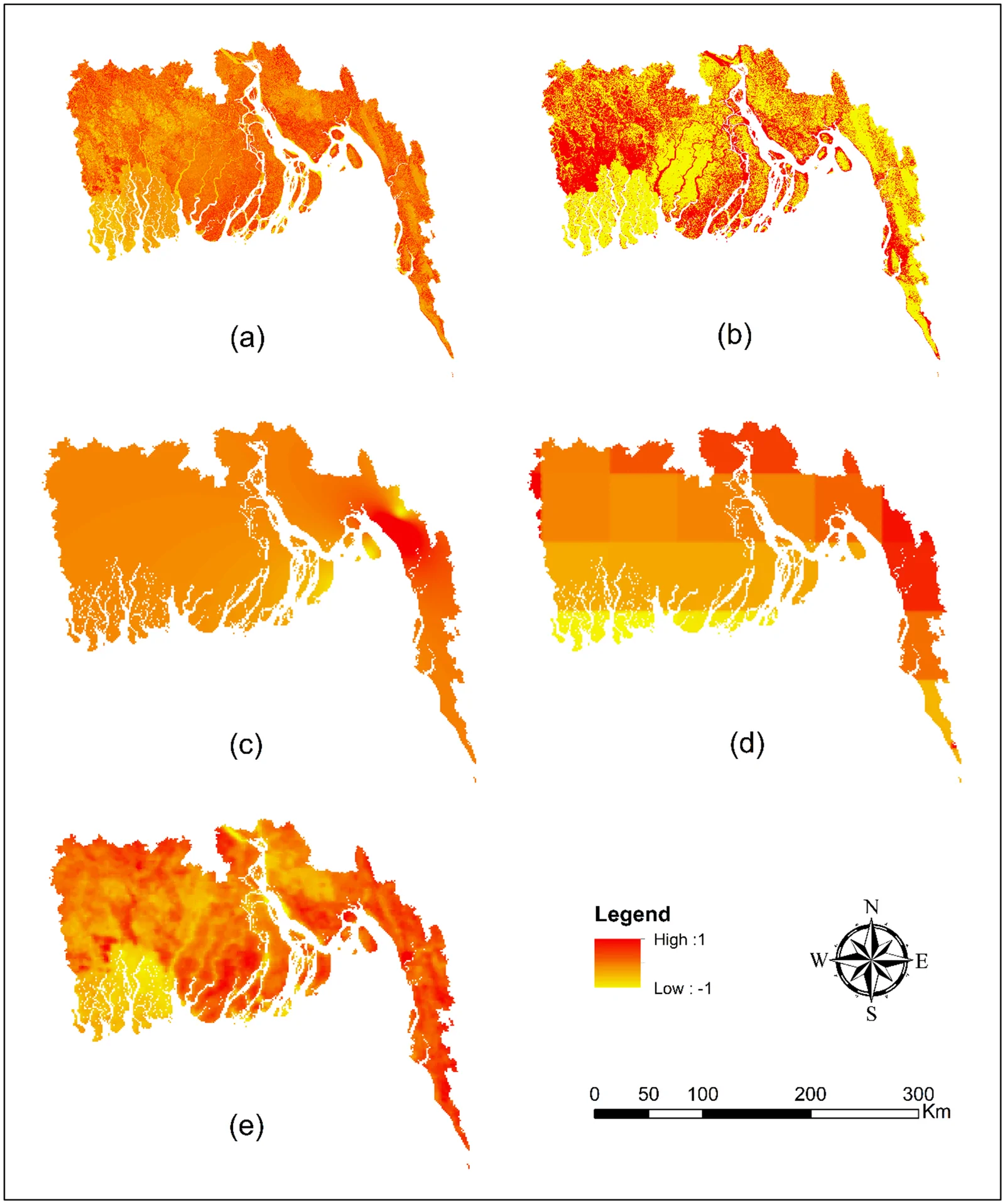
Pressure indicators of 2013: (a) NDBI, (b) Deforestation Index, (c) Cyclone Prone Areas, (d) Presence of Air Pollutants (CO) and (e) LST. Extracted From: (a), (b) & (e) Table 1 (c) RSMC (d) Giovanni (See Table 1).

The pressure indicators of 2013 show variety of extend over the coastal zone. The NDBI was high over every area of the coastal zone except the Sundarbans (Fig 4(a)) The deforestation index was high over the upper part of the Sundarbans and some southern part of coastal zone. (Fig 4(b)). The cyclone prone zone (Fig 4(c)) was high on the east part of the coastal zone but moderately low over the rest part. The air pollutants areas were high on the east part as well (Fig 4(d)) which is mainly Chittagong district and the LST was scattered high over the whole coastal zone except the Sundarbans (Fig 4(e)). (Fig 4)

**Fig 5:**
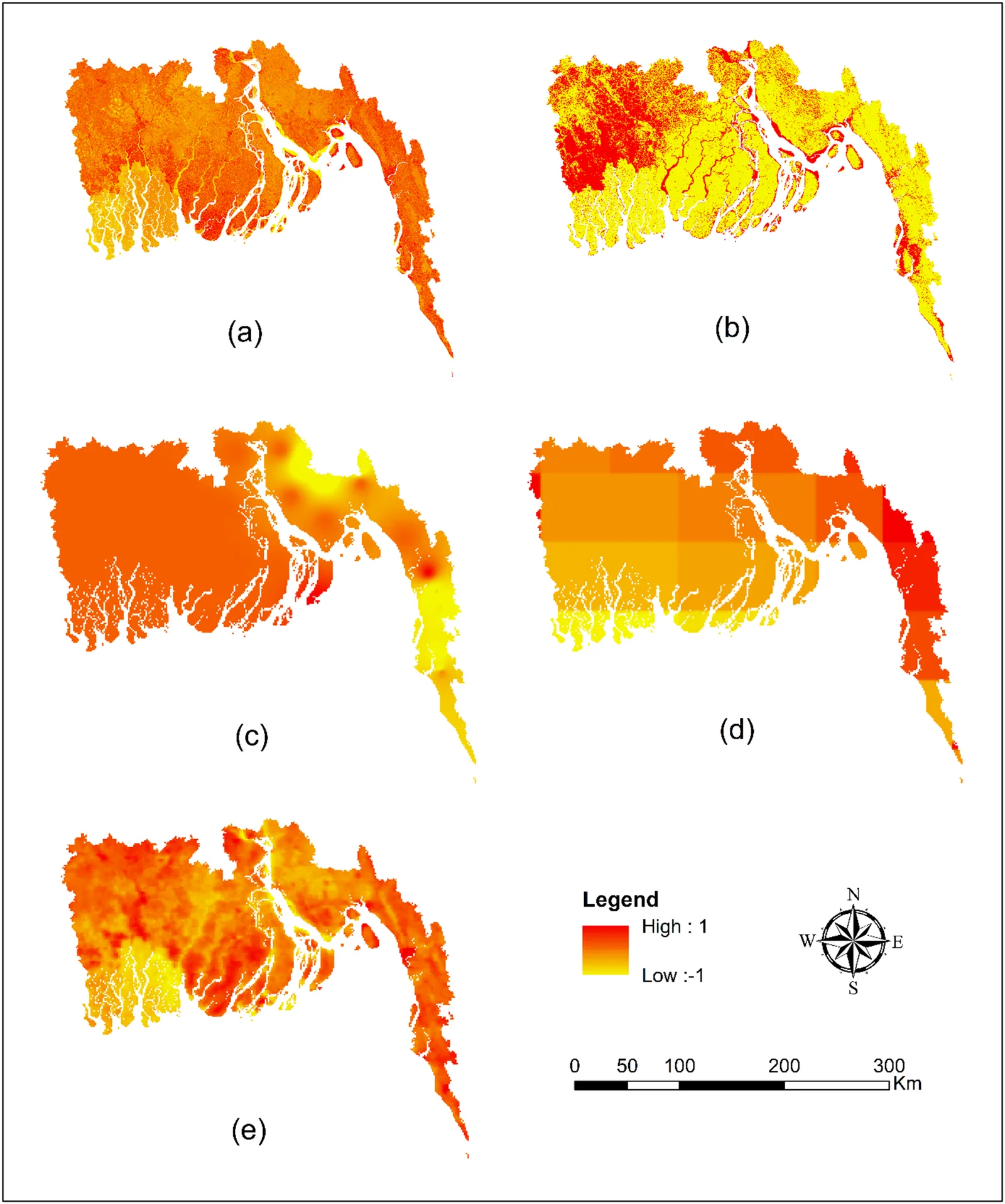
Pressure indicators of 2023: (a) NDBI, (b) Deforestation Index, (c) Cyclone Prone Areas, (d) Presence of Air Pollutants (CO) and (e) LST. Extracted From: (a), (b) & (e) Table 1 (c) RSMC (d) Giovanni (See Table 1).

On the other hand, pressure of 2023 has some change of indicator’s intensity but the major changes can be seen over the deforestation index (Fig 5(b)), that the deforestation area was significantly changes on the southern part of the coastal zone and the cyclone prone areas (Fig 5(c)) has also reduced on the east-southern part of the coastal zone and the rest part has increased the intensity. (Fig 5)

**Fig 6:**
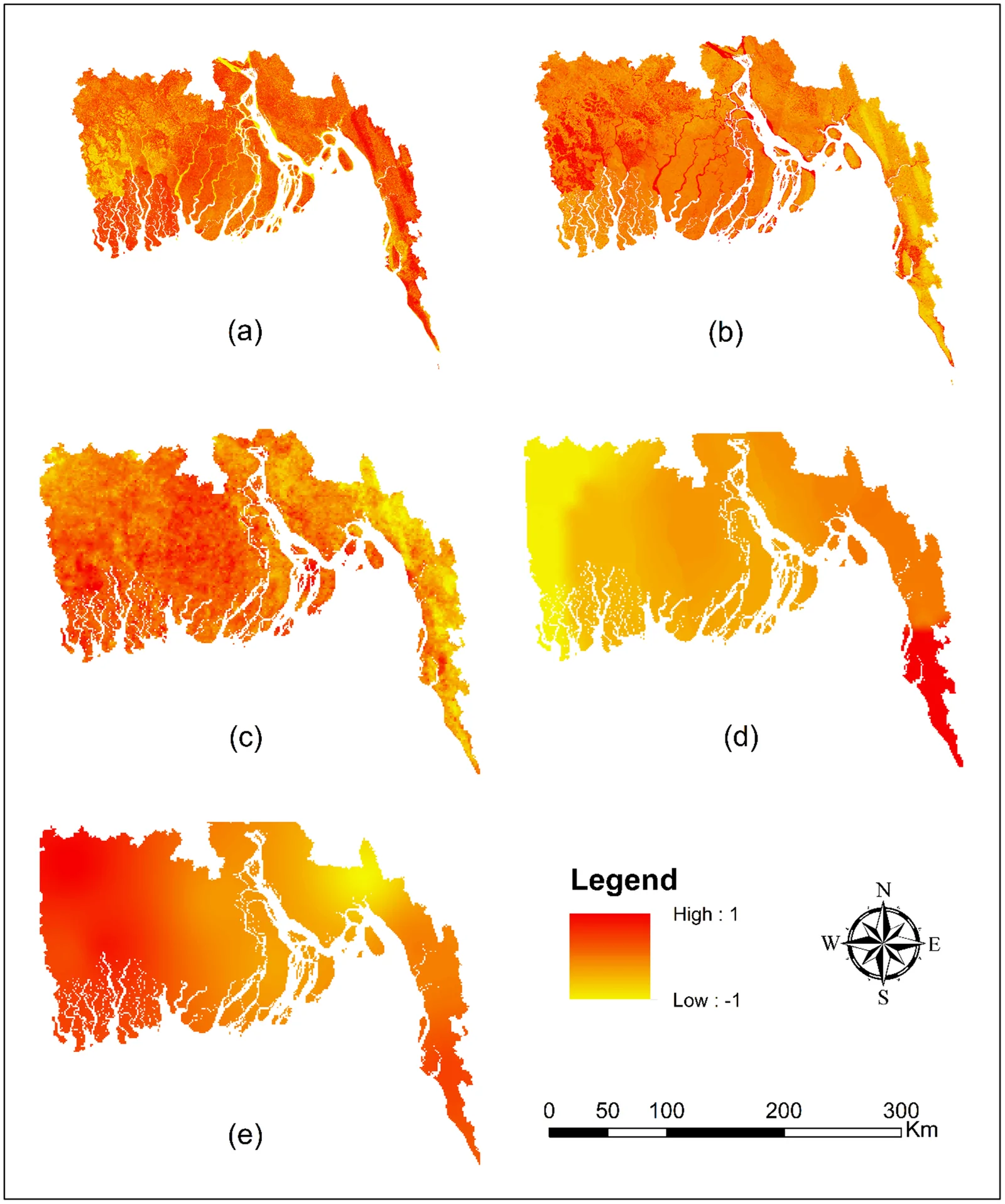
State indicators of 2013: (a) NDVI, (b) NDWI, (c) Soil pH, (d) Precipitation, (e) Temperature rate, (f) Solar radiation, (g) Humidity, (h) Evaporation, (i) Surface wind speed and (j) Ground water storage. Extracted From: (a) & (b) Table 1 (c) SoilGrids250m 2.0 (d), (e), (f) & (g) https://apps.barc.gov.bd/climate/, (h), (i) & (j) Giovanni (See Table 1).

**Fig 7:**
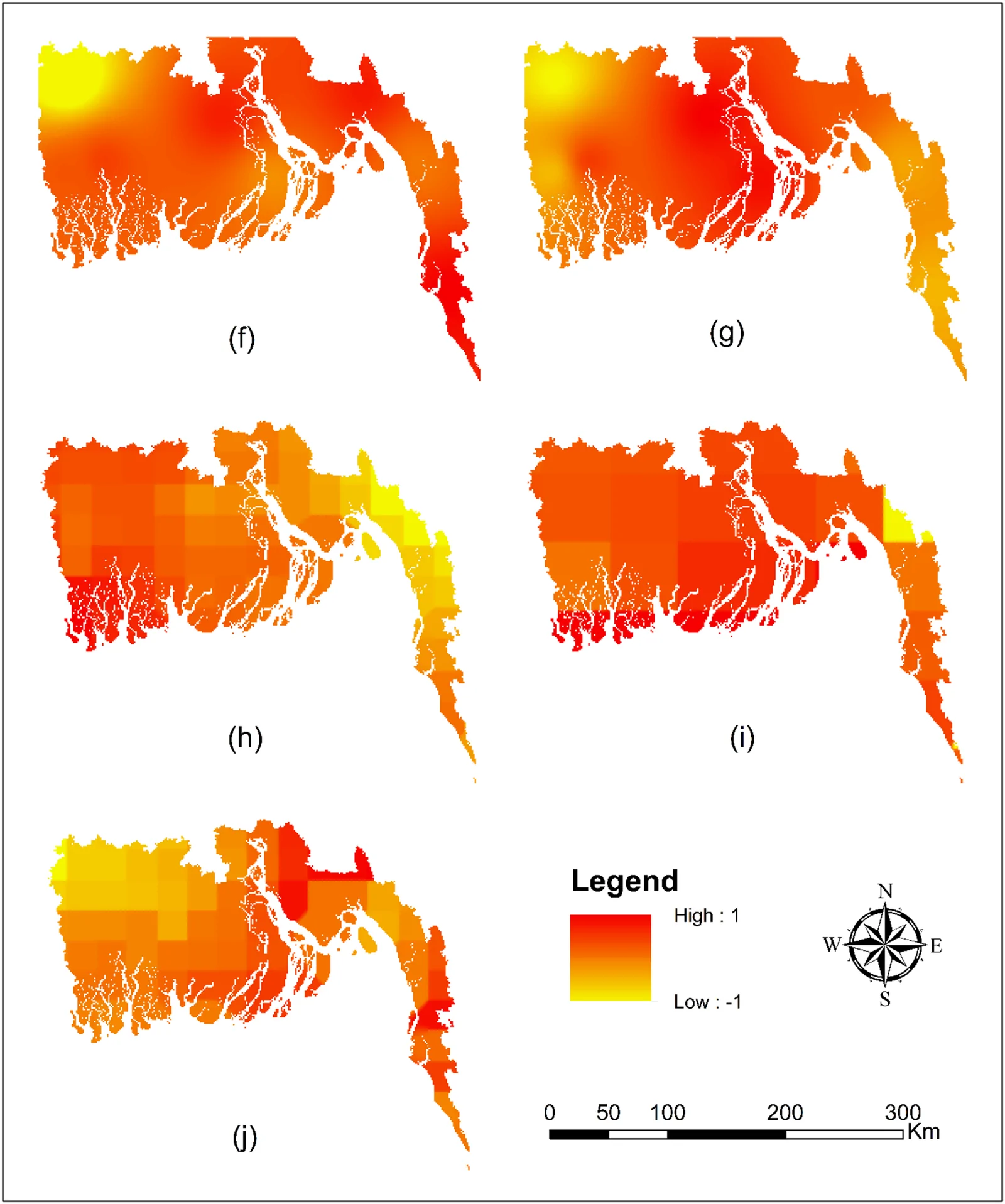
State indicators of 2013: (a) NDVI, (b) NDWI, (c) Soil pH, (d) Precipitation, (e) Temperature rate, (f) Solar radiation, (g) Humidity, (h) Evaporation, (i) Surface wind speed and (j) Ground water storage. Extracted From: (a) & (b) Table 1 (c) SoilGrids250m 2.0 (d), (e), (f) & (g) https://apps.barc.gov.bd/climate/, (h), (i) & (j) Giovanni (See Table 1).

The state indicators of 2013 also have a variety of extends that the NDVI (Fig 6(a)) is high on the Sundarbans and the east-northern part of the coastal zone. The NDWI was high over the west-southern part of the coastal zone but significantly low over the west zone (Fig 6(b)). Soil pH rate was also low over the west zone but high over the rest part (Fig 6(c)). The precipitation rate (Fig 6(d)) is significantly high over the east-southern part of the coastal zone which is mainly the Cox’s Bazar district. The temperature rate is high in the west-northern part of the coastal zone where the Jessore, Narail and Satkhira districts (Fig 6(e)). The solar radiation (Fig 7(f)) and humidity (Fig 7(g)) high over the east part mainly but significantly low on the west-northern part of Jessore district. The evaporation (Fig 7(h)) and surface wind speed (Fig 7(i)) have some scattered extend over the coastal zone but were moderately low over the east part. Groundwater storage (Fig 7(j)) is high over the east-northern and west-northern parts. (Figs 6 and 7)

**Fig 8:**
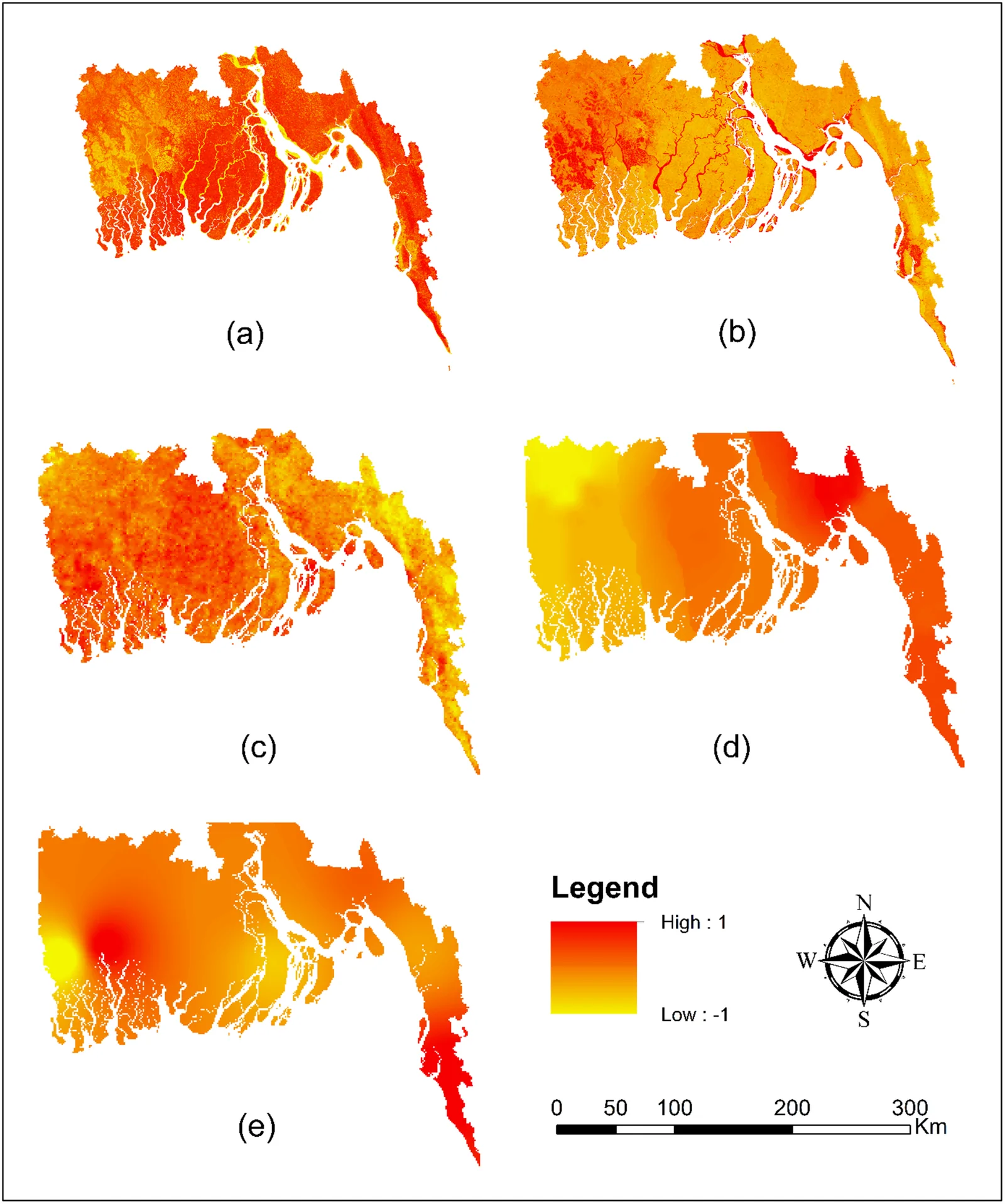
State indicators of 2023: (a) NDVI, (b) NDWI, (c) Soil pH, (d) Precipitation, (e) Temperature rate, (f) Solar radiation, (g) Humidity, (h) Evaporation, (i) Surface wind speed and (j) Ground water storage. Extracted from: (a) & (b) Table 1 (c) SoilGrids250m 2.0 (d), (e), (f) & (g) https://apps.barc.gov.bd/climate/, (h), (i) & (j) Giovanni (See Table 1).

**Fig 9:**
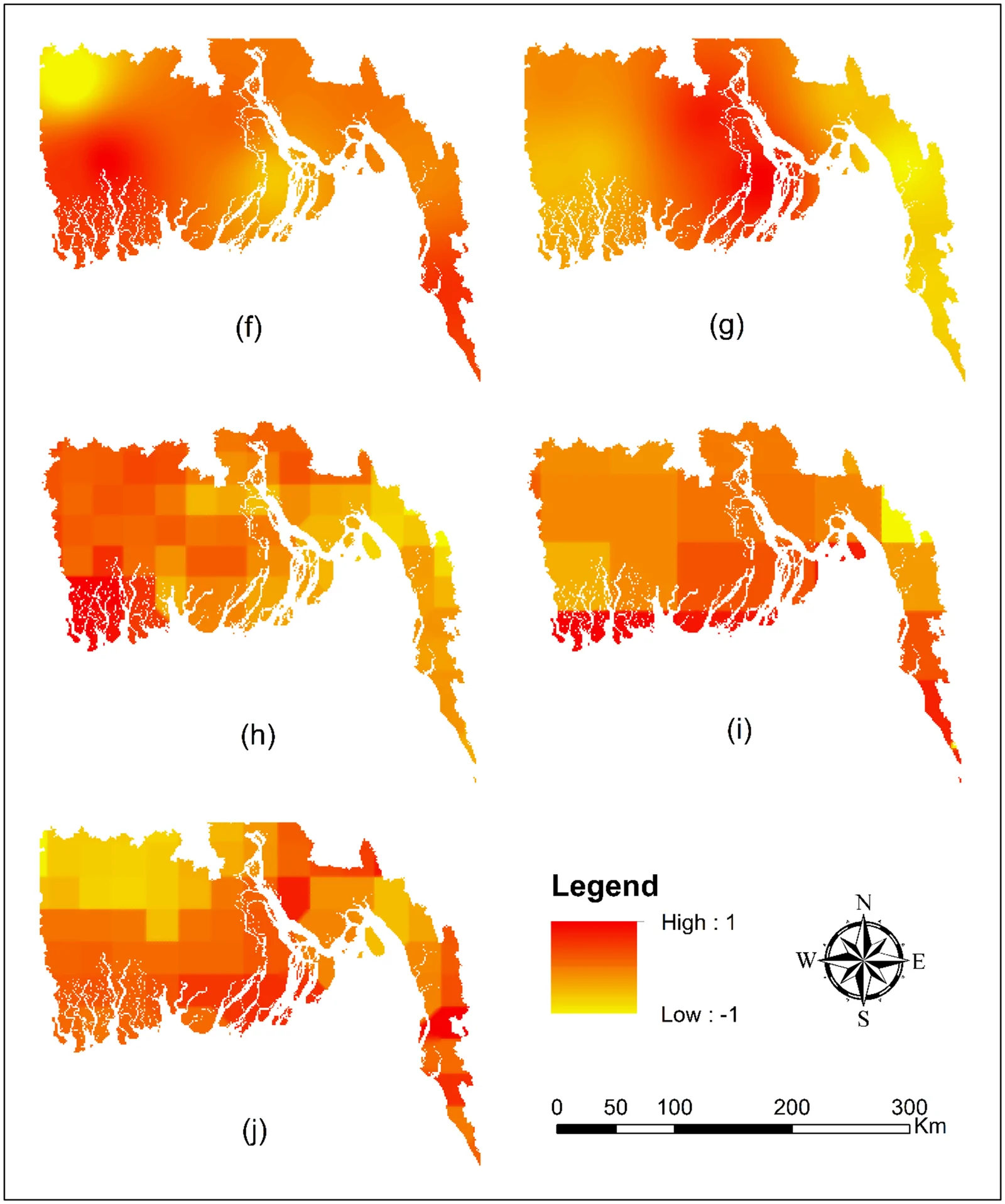
State indicators of 2023: (a) NDVI, (b) NDWI, (c) Soil pH, (d) Precipitation, (e) Temperature rate, (f) Solar radiation, (g) Humidity, (h) Evaporation, (i) Surface wind speed and (j) Ground water storage. Extracted from: (a) & (b) Table 1 (c) SoilGrids250m 2.0 (d), (e), (f) & (g) https://apps.barc.gov.bd/climate/, (h), (i) & (j) Giovanni (See Table 1).

There was some change also in the state indicators intensity of 2023. The NDVI (Fig 8(a)) was low mainly over the west-northern part, the upper part of Satkhira, Bagerhat and Khulna district but it became high on the rest of the coastal zone which shows a significant change than 2013. The NDWI (Fig 8(b)) was high over the west-northern part, the upper part of Satkhira, Khulna and Bagerhat district. The soil pH did not show such kind of significant change than 2013 (Fig 8(c)). The precipitation rate became high (Fig 8(d)) over the east-northern part with addition to the condition of 2013. Higher significant temperature rate has shown over the east-southern part which is the Cox’s Bazar district (Fig 8(e)). On the other hand, the solar radiation (Fig 9(f)) and humidity (Fig 9(g)) did not show such significant change but the humidity increased on the middle part. The evaporation (Fig 9(h)) rate became high over the Sundarbans, the west-southern part and the surface wind speed rate (Fig 9(i)) became low over the major part of the coastal zone except the south part. At last, the groundwater storage (Fig 9(j)) did not show such a significant change. (Figs 8 and 9)

**Fig 10:**
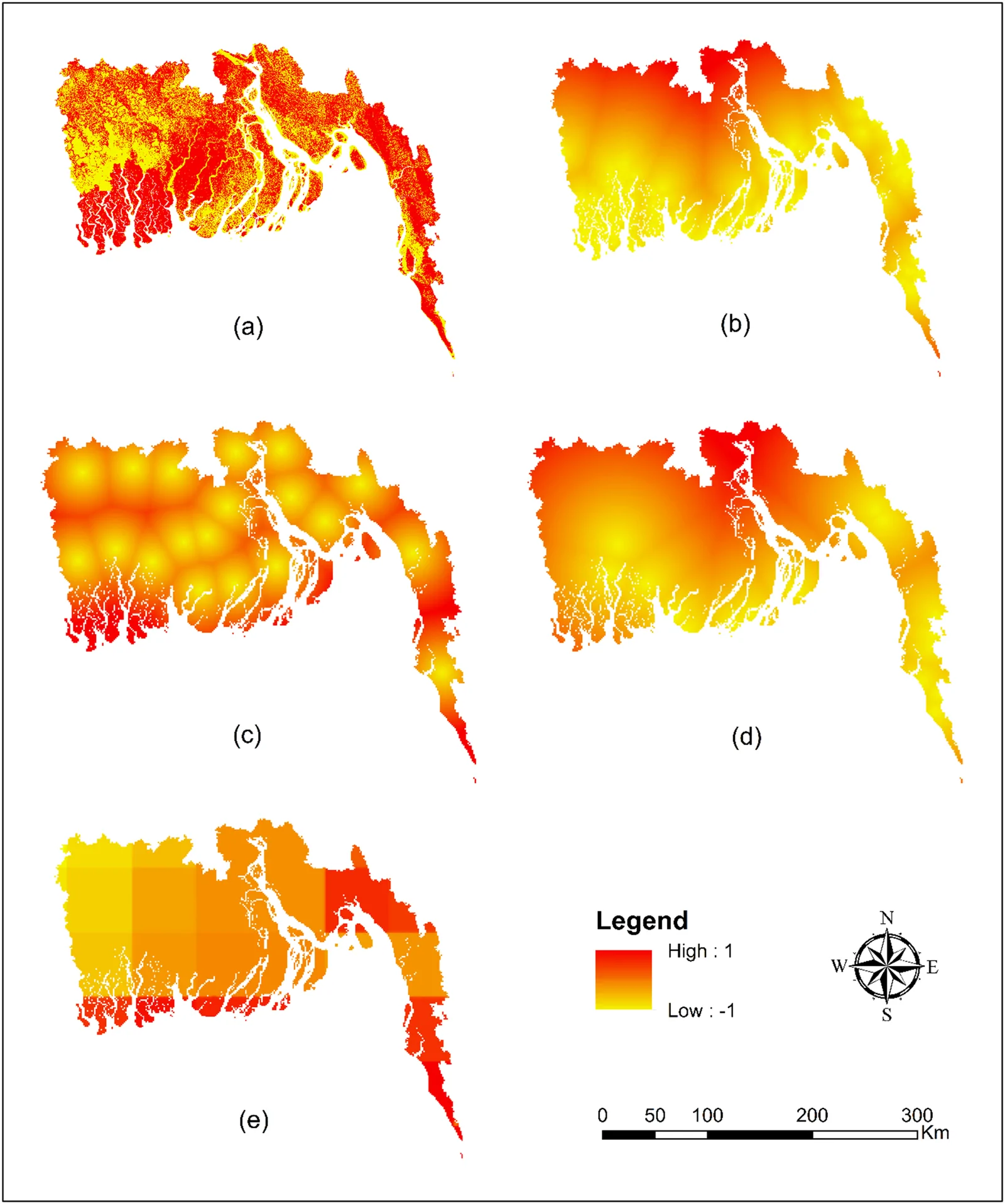
Response indicators of 2013: (a) Afforestation index, (b) Area ratio of natural reserve, (c) Renewable energy potential areas, (d) Spatially protected areas and (e) Latent energy flux. Extracted From: (a) Table 1 (b) & (c) From [17] (d) From [18] and (e) Giovanni (See Table 1).

The afforestation index (Fig 10(a)) of response of 2013 indicated that it had higher rate over the west and east-southern and middle part of the coastal zone but had low rate over the north part and some areas of south part. The higher area ratio of natural reserve (Fig 10(b)) and the spatially protected area (Fig 10(d)) was over the northern part and the renewable energy potential area (Fig 10(c)) extension was higher in the Sundarbans region and the east-southern part of the coastal zone. The latent energy flux rate (Fig 10(e)) was high in the south part and some parts of the east-northern of coastal zone. (Fig 10)

**Fig 11:**
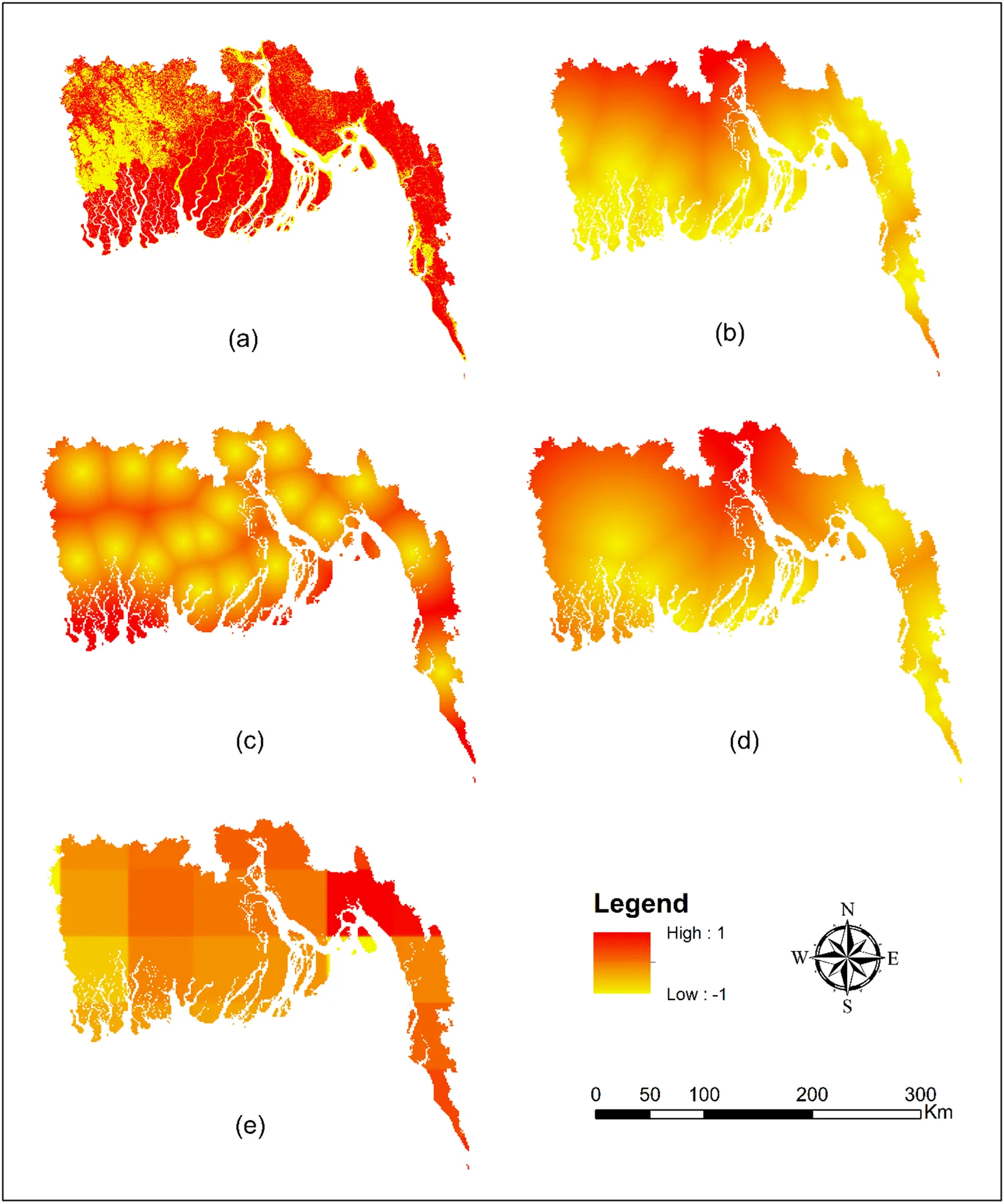
Response indicators of 2023: (a) Afforestation index, (b) Area ratio of natural reserve, (c) Renewable energy potential areas, (d) Spatially protected areas and (e) Latent energy flux. Extracted From: (a) Table 1 (b) & (c) From [17] (d) From [18] and (e) Giovanni (See Table 1).

Finally, with limited changes in the response indicators in 2023, the afforestation index (Fig 11(a)) changed to high over the middle southern, east-northern and southern part of the coastal zone. The latent energy flux (Fig 11(e)) reduced and remained high only in the east-northern part. (Fig 11)

### 3.2 Output of MCA and fuzzification

**Fig 12:**
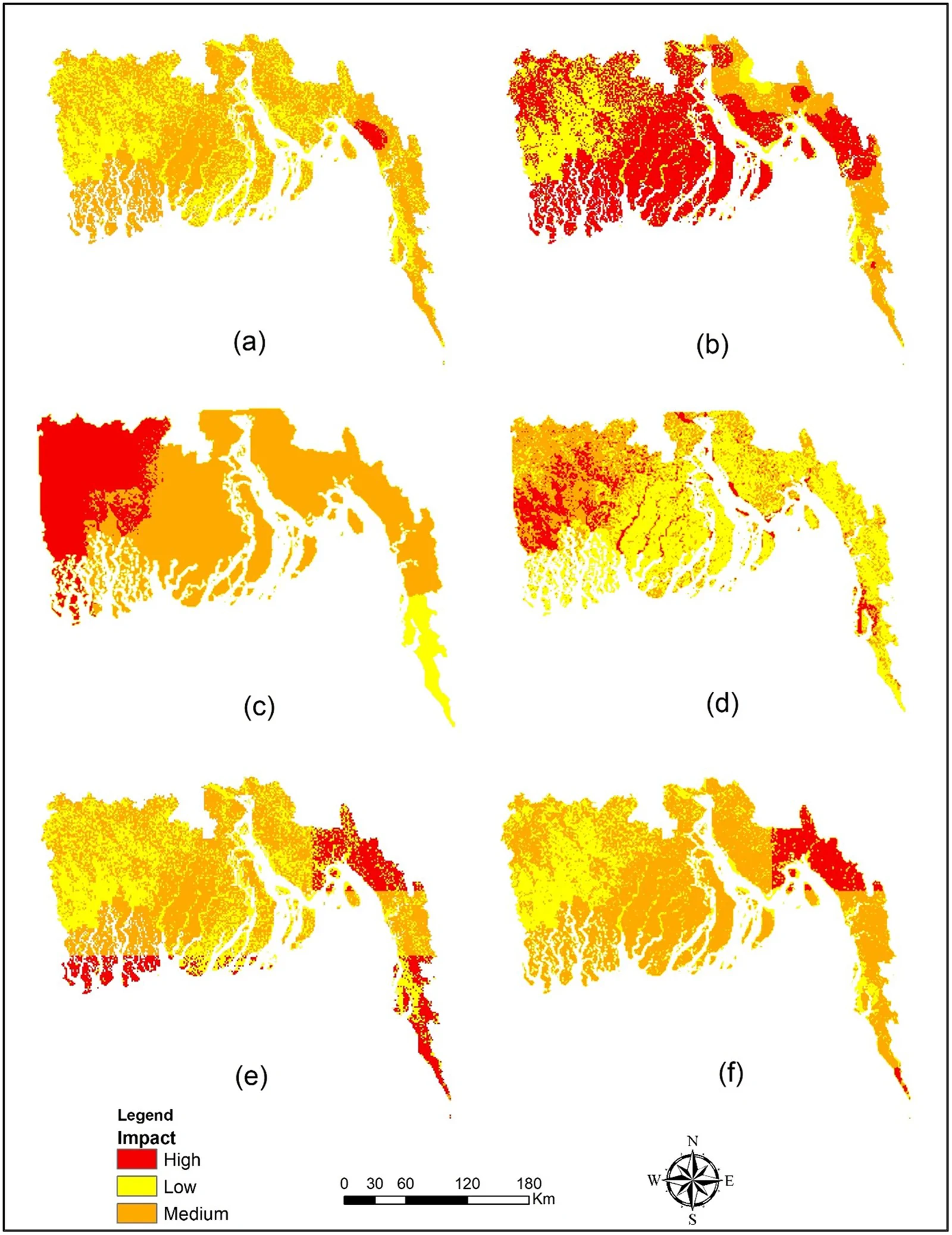
PSR conditions of 2013 and 2023: (a) Pressure 2013, (b) Pressure 2023, (c) State 2013, (d) State 2023, (e) Response 2013, (f) Response 2023. Extracted From: (See Table 1) and previously mentioned extracted data.

**Fig 13:**
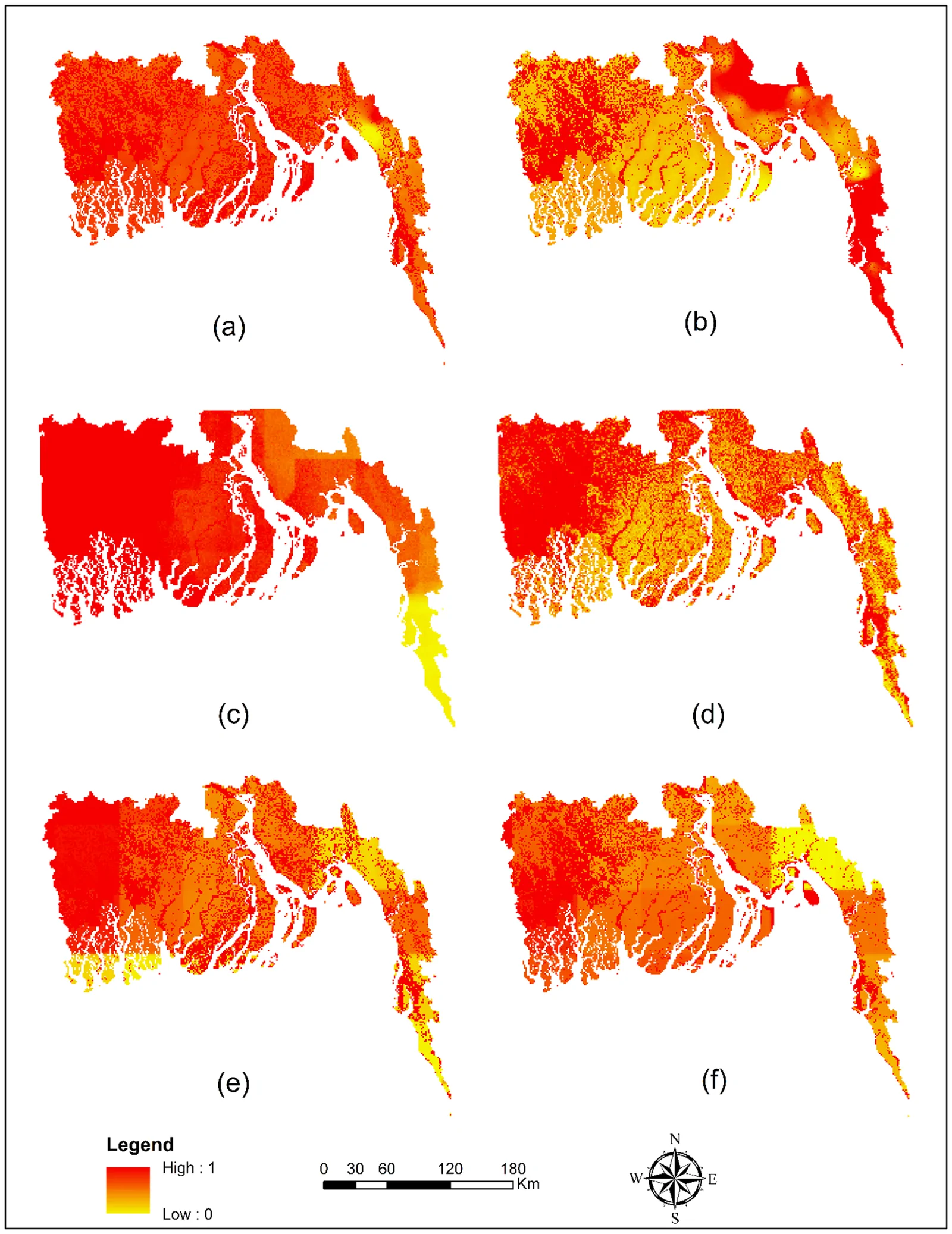
Fuzzification of PSR of 2013 and 2023: (a) Pressure 2013, (b) Pressure 2023, (c) State 2013, (d) State 2023, (e) Response 2013, (f) Response 2023. Extracted From: (See Table 1) and previously mentioned extracted data.

The MCA clearly shows the past and present condition of the pressure, state and response. It clearly visualizes that the pressure condition of 2013 (Fig 12(a)) was good in that the high environmental pressure was only on some small parts of the region that is over the Chittagong district. But in 2023, the pressure condition (Fig 12(b)) was dramatically changed than of 2013. It shows the high environmental pressure extends over the whole coastal zone of Bangladesh except some of the west and east-northern and southern parts. On the other hand, the state condition is moderately reversed to the pressure condition. In 2013, the high state was only some west-northern part, the medium state extended over the major part of the coastal zone and the low state extended over the east-southern part, the Cox’s Bazar district (Fig 12(c)) But in 2023, it became a low state condition over the major part of the coastal zone and this stands the drastic change over the decade (Fig 12(d)). Again, the response condition became lower in 2023 than in 2013, as depicted in (Fig 12(e)) and (Fig 12(f)). (Fig 12). Finally, the fuzzification process which converts the MCA value from 0 to 1 is depicted on (Fig 13).

### 3.3 Ecosystem health condition over the decades

**Fig 14:**
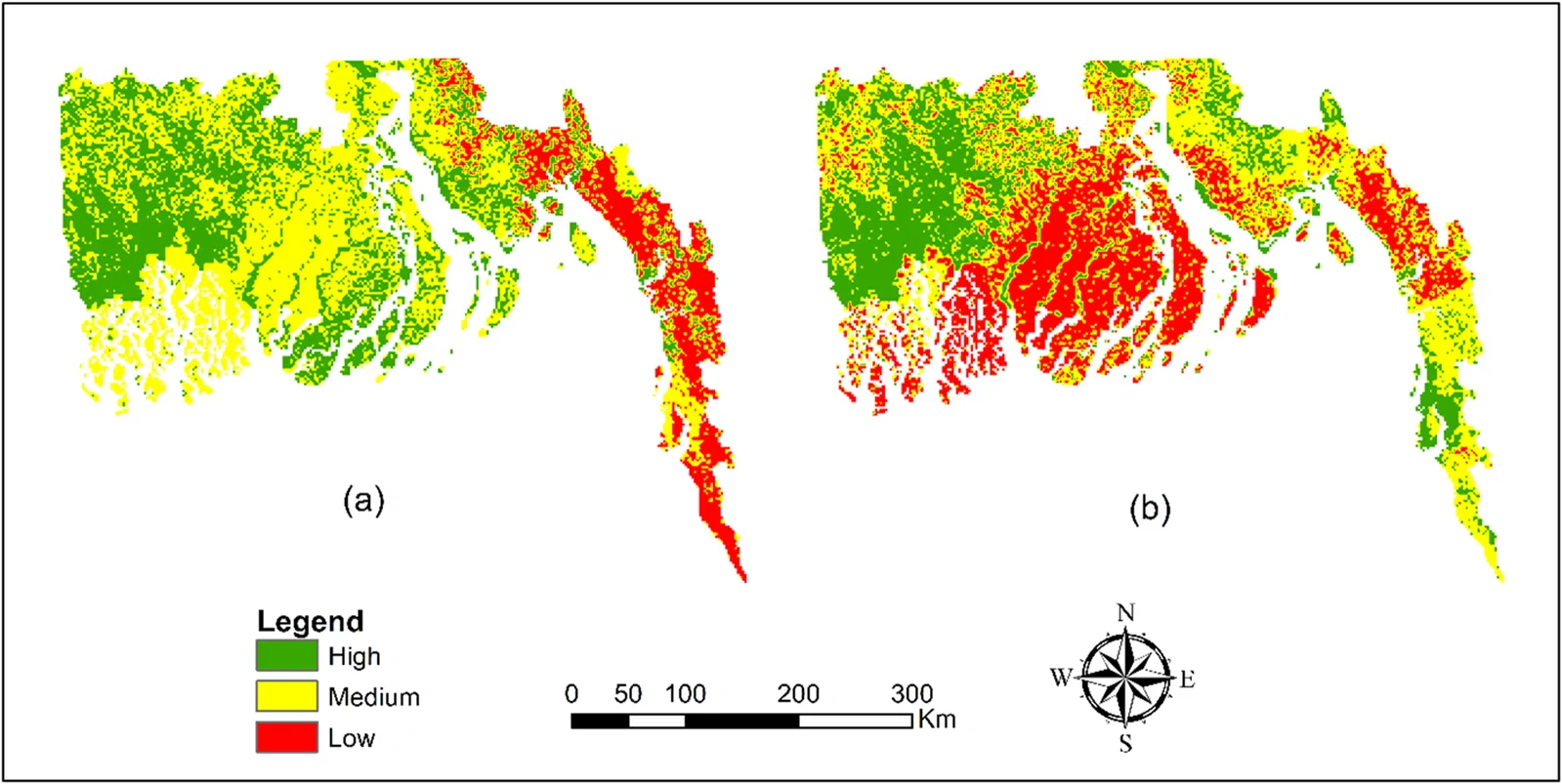
Ecosystem Health Condition: (a) 2013, (b) 2023. Extracted from: (See Table 1) and previously mentioned extracted data.

The EH highlights (Fig 14) a significant increase in areas of low EH over a decade. This indicates a decline in biodiversity. From the (Fig 14(a)), in 2013 the low EHC mostly was in the east part of the coastal zone whereas, in 2023, it spread over the whole coastal region mostly over the middle southern part (Fig 14(b)),. The rest region belonged to medium and high EH in 2013 but the medium EH significantly decreased in 2023. There have been also noticeable changes in high EH over the years. (Fig 14)

**Fig 15:**
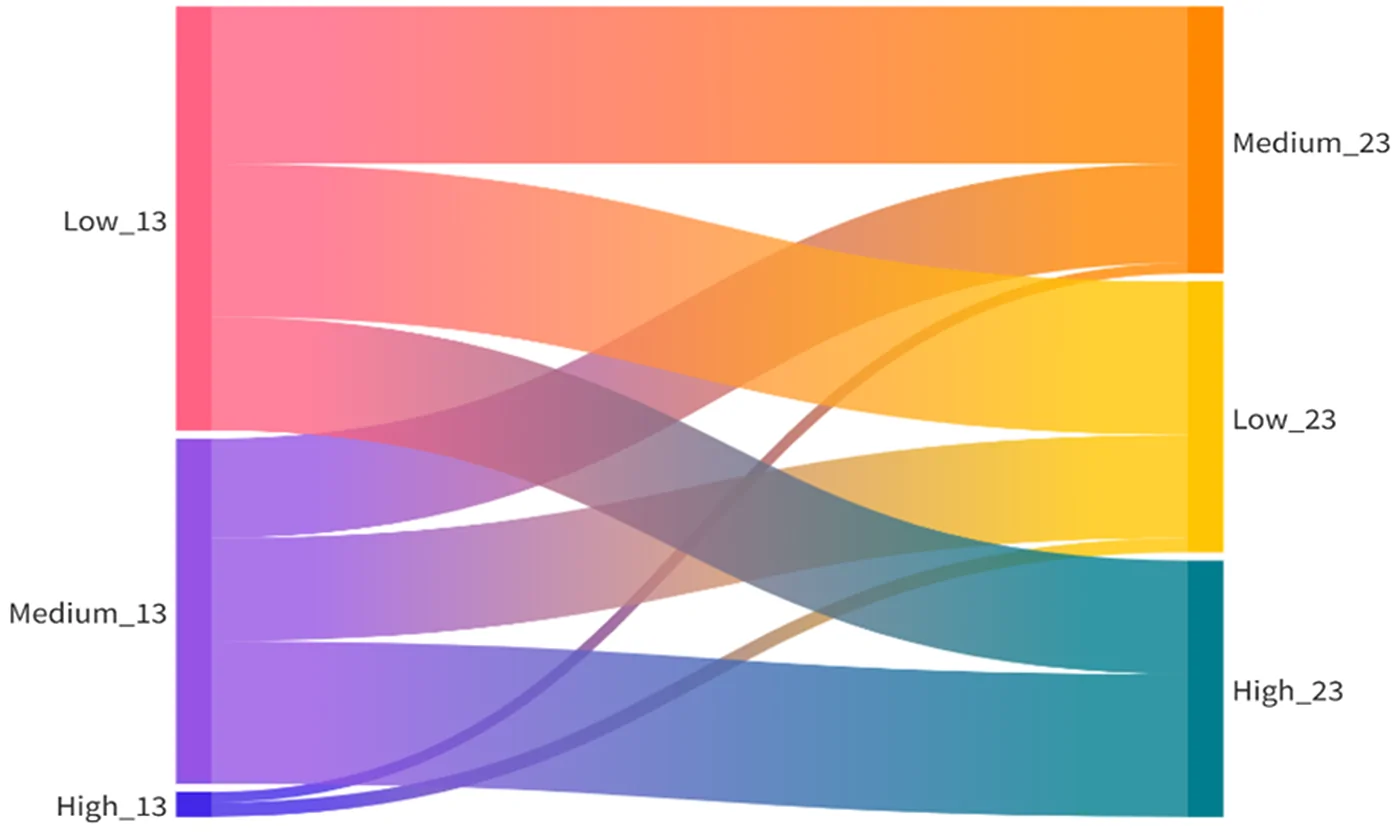
Change detection through Sankey Diagram. Extracted from: Table 1 and previously mentioned extracted data.

This diagram (Fig 15) visualizes that, how the high, medium and low EHC became changed over the decades. The EHC deteriorates mostly in 2023.

The overall change detection was assessed through three distinct levels that are in high, medium and low (Table 2)*.* In 2013, areas of 36.17% of the coastal zone were classified as high. 48.18% of areas fell into the medium category and 15.11% of coastal zone areas were categorized as low EH. On the other hand, in 2023, the areas classified as high health turned into 29.79%; a decrease of 6.92%. The areas which were categorized as medium turned into 35.34%, which also decreased by 12.84%. But there has been a significant increase (19.76%) of low EH areas which turned into 34.87%.

**Table 2:** EHC trend of 2013 and 2023.

Status	EHC 2013%	EHC 2023%	Change %
High	36.71	29.79	−6.92 *
Medium	48.18	35.34	−12.84 *
Low	15.11	34.87	+19.76 *

*The minus value indicates degradation and plus value indicates amelioration.

**Fig 16:**
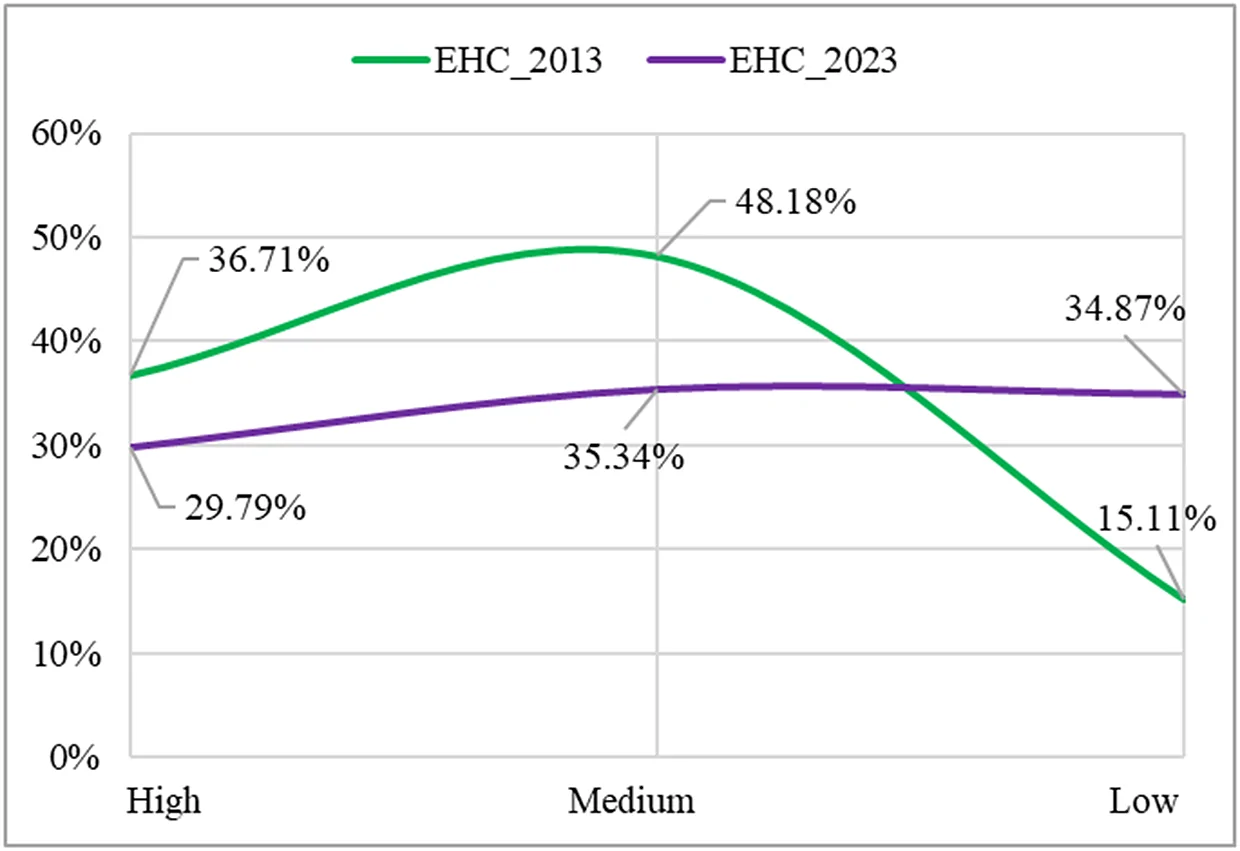
Visual representation of EH changing trend. Extracted from: Extracted data using (Fig 14).

It clearly visualizes that the high EH condition deteriorates mostly in 2023; which is about 6.92% of the area. This graph (Fig 16) represents the downward trend of EH in 10 years. It shows a significant decrease of medium EH; in addition, with a more significant increase of low EH by 2023.

### 3.4 Ecosystem health change detection of 2013

Area of High EHC belongs to (Table 3) Satkhira district. After this appears Khulna, Patuakhali, Jessore, Gopalganj, Lakshmipur, Narail, Noakhali, Bhola, Bagerhat, Barguna, Chandpur, Feni, Barisal, Shariatpur, Chittagong, Pirojpur, Jhalokati and Cox’s Bazar has no high EHC.

**Table 3:** Change detection of 2013.

District	High %	Low %	Medium %
Bagerhat	37.29	–	62.71
Barguna	34.44	–	65.56
Barisal	26.08	–	73.92
Bhola	37.75	–	62.25
Chandpur	34.20	23.19	42.61
Chittagong	19.81	66.85	13.34
Cox’s Bazar	–	61.99	38.01
Feni	32.09	63.23	4.68
Gopalganj	45.93	–	54.07
Jessore	49.45	–	50.55
Jhalokati	7.24	–	92.76
Khulna	54.25	–	45.75
Lakshmipur	42.47	14.40	43.13
Narail	42.32	–	57.68
Noakhali	40.45	23.16	36.39
Patuakhali	51.69	–	48.31
Pirojpur	18.50	–	81.50
Satkhira	60.68	–	39.32
Shariatpur	22.71	–	77.29

Medium EHC belongs to (Table 3) at most Jhalokati district. The rest are Pirojpur, Shariatpur, Barisal, Barguna, Bagerhat, Bhola, Narail, Gopalganj, Jessore, Patuakhali, Khulna, Lakshmipur, Chandpur, Satkhira, Cox’s Bazar, Noakhali, Chittagong and Feni district.

The Low EHC belongs to (Table 3) Chittagong district. The rest low EHC are Feni, Cox’s Bazar, Chandpur, Noakhali, Lakshmipur district.

On the other hand, Bagerhat, Barguna, Barisal, Bhola, Gopalganj, Jessore, Jhalokati, Khulna, Narail, Patuakhali, Pirojpur, Satkhira district has no low EHC.

### 3.5 Ecosystem health change detection of 2023

District of High EHC (Table 4) belong to Narail in 2023. The other district of high EHC is Khulna, Satkhira, Jessore, Gopalganj, Cox’s Bazar, Bagerhat, Shariatpur, Chandpur, Noakhali, Feni, Lakshmipur, Barisal, Pirojpur, Chittagong, Patuakhali, Bhola, Barguna, Jhalokathi.

**Table 4:** Change detection of 2023.

District	High %	Low %	Medium %
Bagerhat	32.62	47.13	20.25
Barguna	5.17	84.31	10.52
Barisal	14.99	57.79	27.22
Bhola	5.94	77.82	16.24
Chandpur	28.07	10.25	61.68
Chittagong	11.76	36.31	51.93
Cox’s Bazar	39.96	1.57	58.46
Feni	19.39	15.03	65.58
Gopalganj	41.38	18.97	39.65
Jessore	41.54	9.74	48.72
Jhalokathi	5.12	84.68	10.20
Khulna	62.40	12.10	25.50
Lakshmipur	18.80	31.33	49.87
Narail	63.47	5.21	31.32
Noakhali	26.51	23.75	49.74
Patuakhali	9.12	71.86	19.02
Pirojpur	12.55	69.58	17.87
Satkhira	57.85	15.74	26.40
Shariatpur	30.10	26.12	43.78

The medium EHC belongs to (Table 4) Feni. The others are Chandpur, Cox’s Bazar, Chittagong, Lakshmipur, Noakhali, Jessore, Shariatpur, Gopalganj, Narail,Barisal, Satkhira, Khulna, Bagerhat, Patuakhali, Pirojpur, Bhola, Barguna and Jhalokathi district.

At most, low EHC has (Table 4) Jhalokathi district. Others are Barguna, Bhola, Patuakhali, Pirojpur, Barisal, Bagerhat, Chittagong, Lakshmipur, Shariatpur, Noakhali, Gopalganj, Satkhira, Feni, Khulna, Chandpur, Jessore, Narail and Cox’s Bazar district.

### 3.6 Validation of the result

#### 3.6.1 The ROC curve of 2013.

**Fig 17:**
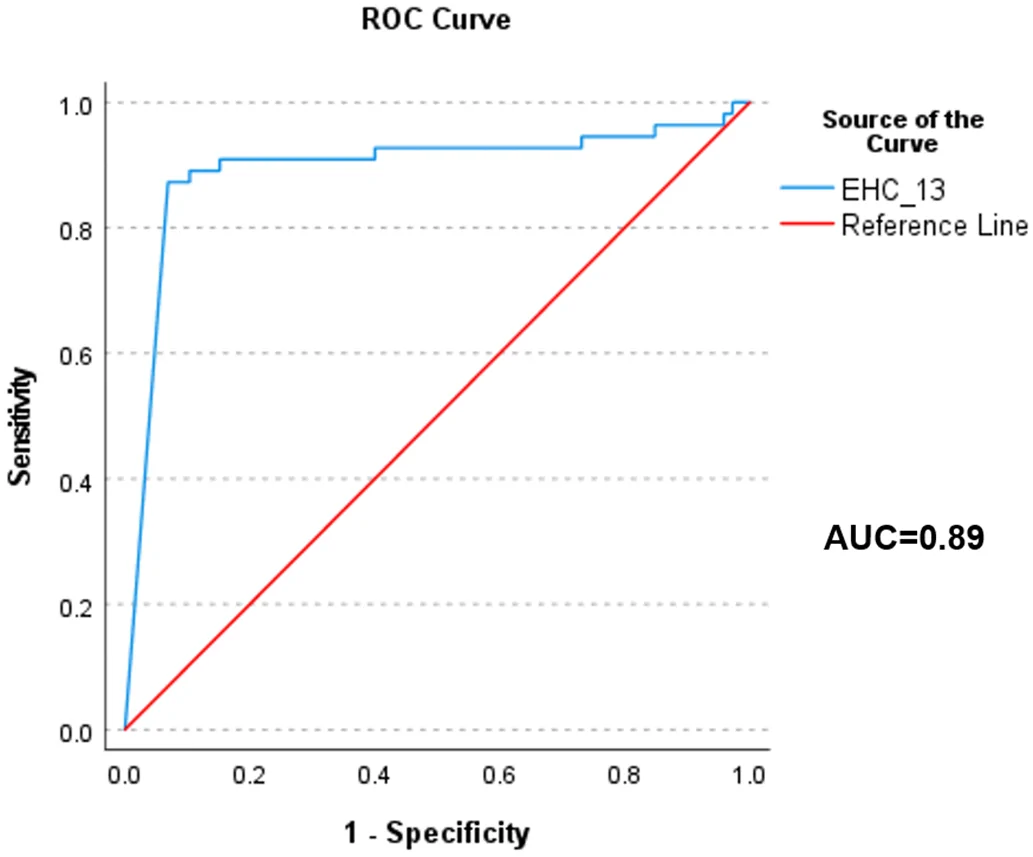
AUC and ROC analysis of 2013. Extracted from: Extracted data using (Fig 14).

The Area Under Curve (AUC) for the RF model has been found 0.89 (Fig 17). That means this machine learning model performs well for assessing the EH of 2013. The standard error is 0.023 which is statistically significant at a 95% confidence level. So, the RF machine learning model results are mostly accurate for assessing EH of 2013.

#### 3.6.2 The ROC curve of 2023.

**Fig 18:**
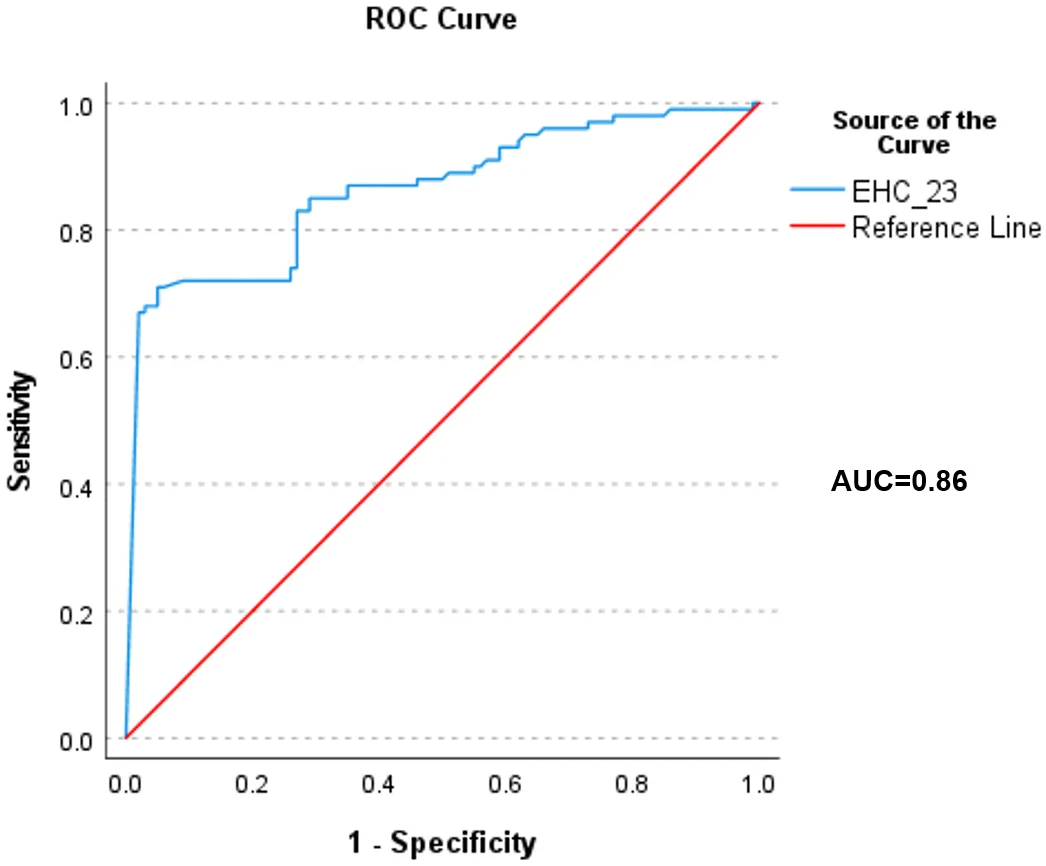
AUC and ROC analysis of 2023. Extracted from: Extracted data using (Fig 14).

The Area Under Curve (AUC) for the RF model has been found 0.86 (Fig 18). That is the machine learning model performs well for assessing the EH of 2023. The standard error is 0.026 which is statistically significant at a 95% confidence level. So, the RF machine learning model results are mostly accurate for assessing EH of 2023.

## 4. Discussion

The study captured the EH of the coastal zone of Bangladesh by incorporating random forest machine learning model with remote sensing and GIS. This reveals that the EH mainly degraded over the decades between 2013 and 2023. Basically, the low ecosystem existed in the southern-east part of Bangladesh consisting of the district of Chittagong, Cox’s Bazar, Feni and Noakhali in 2013. But, in 2023, it revealed a horrible scenario of the coastal zone of Bangladesh that, the low ecosystem extends over the whole southern part, and it covers the middle part as well. The high EH was 36.71% of the area in 2013 but it decreased 6.92% in 2023 that results in 29.79%. The medium ecosystem was 48.18% in 2013 but it decreased 12.84% in 2023 which results in an area of 35.34% as well. Here the startling thing is that the low EH increased by 19.76% resulting in 15.11% to 34.87% over the decades. This makes the matter of concern that how the ecosystem degraded over the past 10 years. In 2013, the high ecosystem belonged to Satkhira district with 60.68% of the area of that district and after came Khulna district with 54.25% of the area of that district. The medium EH belonged to the Jhalokati district 92.76% of that area. The low EH belonged to the Chittagong district with 66.85% of the district area and after it came Feni 63.23% and Cox’s Bazar 61.99% as well. But in 2023, the high EH contained the district of Narail at 63.47% and Khulna at 62.40%. Medium EH belonged to Feni at 65.58% and low EH had Jhalokathi at 84.68% of the area and Barguna at 84.31% of the area. The ML model was validated through the ROC curve with an AUC of 0.89 in 2013 and an AUC of 0.86 in 2023 which indicates the best fitting of the ML model.

An analysis by [47] on land cover changes and LST dynamics on Cox’s Bazar Between 2013 and 2018, the mixed forest areas experienced a drastic reduction of 97%, while built-up areas grew by 161.78%. This deforestation and urbanization have significantly increased LST, with the cooler zones shrinking to negligible levels and high temperature zones expanding substantially. The study also notes the positive but limited impact of reforestation efforts from 2018 to 2024, which resulted in a 43% increase in green areas. Mixed forest areas were identified as the most effective in reducing LST, emphasizing the critical role of green spaces in mitigating heat stress and stabilizing the local climate which considers the improvement of ecosystem health of Cox’s Bazar in 2023. Previously, an investigation by (Hossain et al., 2016) of the south-western coastal zone of Bangladesh revealed that it has been changed over the decades which were mostly negative. It was found that, various driving force such as fish and food production, transforming to shrimp culture from rice production increases salinity in soil which are the major cause of environmental deterioration. Again, another study was conducted to track the changing ecosystem of Cox’s Bazar, the southern coastline and place of the longest natural sea beach. This study revealed that, besides generating employment and development, economic activities and the nature-dependent livelihood facing threats and increasing risk and exposure and results in vulnerability. The satellite imagery visualizes the land use changing pattern for human reason and the questionnaire survey found out the key factors of degradation of the ecosystem including the fishing, salt shrimping, agriculture, tourism, various business, hotel and restaurant industries, etc. [9]. Due to both natural and man-made factors, Bangladesh’s coastline has experienced significant changes in recent decades. Because Hatiya Island is located at the mouth of the Meghna River, where flows are greatest, it exhibits constant erosion to the north, much like the high shoreline alterations around the Padma. Alluvial islands are more vulnerable as a result of increased erosion caused by rising water levels and changed rainfall [48]. The Kuakata coastline has seen 13.59 km2 of land erosion and 3.27 km2 of coastal land increase between 1989 and 2020. On the other hand, Poor management and uncontrolled land reclamation are the main causes of coastal land loss and degradation. Over the past 30 years, hotels, resorts, and retail centers have grown in Bangladesh’s tourist destinations without keeping an eye on changes in the land cover. [49]. The low-lying topography of Bangladesh and climate change are cited in studies as the primary causes of coastal deterioration, which results in erosion, storm surges, saltwater intrusion, flooding, drought, cyclones, and inundation. Cyclones alone are responsible for 49% of catastrophe fatalities. Shipbuilding, sewage, industrial and domestic waste, and agrochemical pollution all contribute to the degradation of coastal waters. Rising sea surface temperatures (0.05 °C/year), cyclone frequency (26% rise from 1881 to 2001), decreased freshwater flow from dams and shrimp farms, and continuous mangrove destruction are all problems facing the Sundarbans. A few more factors are human accessibility, habitat degradation, overexploitation of marine and coastal resources, unplanned coastal structural development, seashell and coral extraction, unsustainable management, and stakeholder involvement [5].

Though these studies do not assess the EH of the coastal zone of Bangladesh directly using machine learning model conjugating remote sensing and GIS, they do conclude with one thing that, the EH of the coastal region of Bangladesh is declining day by day, which also has been revealed in this study over the decades of 2013 and 2023. In addition, the methodology and technique of assessment of EH of this study stands novel in this field.

With many other things, this study holds some limitations. That’s first of all, this does not cover the whole country. That’s why it cannot make the assumption of EH of the other part of the country. Because there are different land characteristics and areas in the rest of the country. Because the coastal zone and coastal districts’ nature, the environment, and the economy, etc. are different than the other parts of the country. On the other hand, as the investigation visualizes the EH of the coastal zone of Bangladesh, depending on the maximization and minimization of the study area the result may differ. That is, if EH assesses on individual district or some part of the coastal zone; in reason of the data characteristics-that the machine learning model handles. In addition, the change in sample size also might change in result of ecosystem health.

The EH assessment can concern about land and infrastructure allocation and control of usage. The concerning factor can promote green infrastructure development and help to take decision on emerging conditions and natural events. EHC phenomena can direct the policy maker and the authority to take actions for the betterment of the coastal ecosystem on which the major proportion of the life of the country’s depends including human life and the economy added with the country’s welfare.

## 5. Conclusion

This investigation indicates that the overall EH has declined over the decades. It clearly visualizes that the condition is downgrading from time to time. This phenomenon points to an urgent need for conservation and restoration efforts. The biodiversity loss, water quality decline, mangrove forest depletion, fisheries decline, climate vulnerability, etc. has become the major field of declination. As the condition worsens from 2013 to 2023, without taking immediate steps, will degrade more the EH of the coastal zone of Bangladesh in the future. This will then become a threatening situation. Such conditions will affect human health, environmental health and also affect the country, which may result in economic loss. The government of Bangladesh (GoB) has taken a number of steps to conceptualize integrated coastal zone management (ICZM) in Bangladesh to meet the steps to improve the coastal zone ecosystem health. The initiatives are Off-Shore Islands Development Board (1977–1982), Coastal Environment Management Plan for Bangladesh (1987), Special Parliamentary Committee on Coastal Area Development (1988–1990), Coastal Area Resources Development Plan (CARDMA) (1988), National Tourism Policy (1992), National capacity building approach the ICZM initiative (1997), Tsunami vulnerability map (2005), Coastal Zone Policy (CZP) (2005), Coastal development strategy (CDS) (2006), The National Tourism Policy (2009) and Bangladesh Delta Plan (BDP) 2100 [5]. Integrated management, collaboration techniques with public and private, ecosystem restoration, enhancing ecosystem services in a sustainable way, policy reforms, raising awareness and education, public concerns especially to the coastal population are required to lead a rejuvenation of coastal EH, resilience, and healthier future for both nature and the people.
